# From *in vitro* to *in vivo* Models of Bacterial Biofilm-Related Infections

**DOI:** 10.3390/pathogens2020288

**Published:** 2013-05-13

**Authors:** David Lebeaux, Ashwini Chauhan, Olaya Rendueles, Christophe Beloin

**Affiliations:** Institut Pasteur, Unité de Génétique des Biofilms, 25 rue du Dr. Roux, 75724 Paris cedex 15, FRANCE; E-Mails: david.lebeaux@yahoo.fr (D.L.); ashwini.chauhan@pasteur.fr (A.C.); olaya.rendueles@env.ethz.ch (O.R.)

**Keywords:** biofilm, *in vitro* models, surrogate non-mammalian models, tissue-associated biofilm models, device-related biofilm models

## Abstract

The influence of microorganisms growing as sessile communities in a large number of human infections has been extensively studied and recognized for 30–40 years, therefore warranting intense scientific and medical research. Nonetheless, mimicking the biofilm-life style of bacteria and biofilm-related infections has been an arduous task. Models used to study biofilms range from simple *in vitro* to complex *in vivo* models of tissues or device-related infections. These different models have progressively contributed to the current knowledge of biofilm physiology within the host context. While far from a complete understanding of the multiple elements controlling the dynamic interactions between the host and biofilms, we are nowadays witnessing the emergence of promising preventive or curative strategies to fight biofilm-related infections. This review undertakes a comprehensive analysis of the literature from a historic perspective commenting on the contribution of the different models and discussing future venues and new approaches that can be merged with more traditional techniques in order to model biofilm-infections and efficiently fight them.

## 1. Introduction

**Figure 1:**
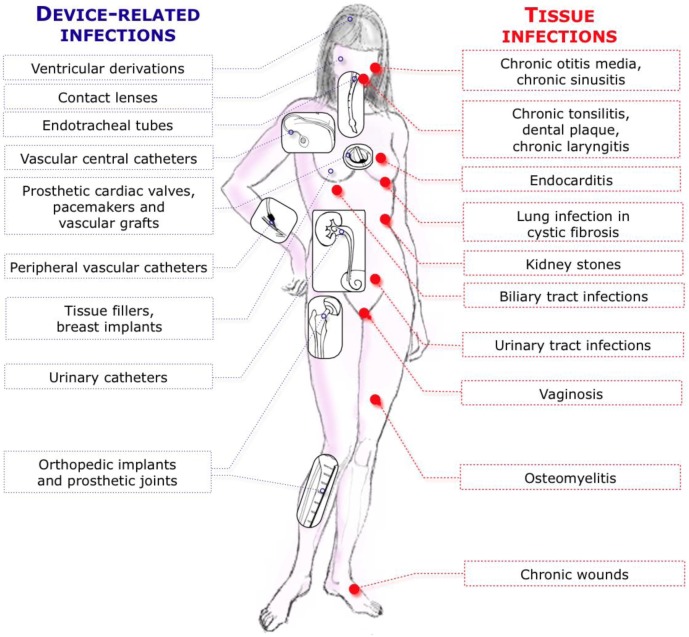
Most studied biofilm-related infections in humans. Adapted from [17].

Pioneer studies by A.T. Henrici in the early 20th century [1] and later by J. W. Costerton and colleagues [2,3] have pointed to the existence of microorganism populations living on surfaces. Nowadays it is well accepted that, in most environments, microorganisms can switch from a free-living state to a sessile mode of life to form biofilms displaying specific properties. Among these specific properties is an enhanced tolerance to all sort of adverse conditions including desiccation and high concentrations of antimicrobial agents such as biocides, antibiotic and antifungal compounds [4,5,6,7,8]. Microorganisms growing and persisting on surfaces are problematic because, on one hand, they represent a source of contamination when present in a closed hospital environment and, on the other hand, when introduced into the body, they can develop on medical devices or tissues such as mucosa to form antimicrobial tolerant biofilms. N. Hoiby, J.W. Costerton and their collaborators were the first to suspect a direct correlation between bacteria growing as communities and persistent infections notably in the case of *Pseudomonas aeruginosa* colonizing the lungs of cystic fibrosis patients [9,10,11,12,13,14]. Since then, an increased awareness of the link between microorganisms growing on surfaces and development of human infections led to the estimation that 65% (Centers for Disease Control and Prevention/CDC [15]) to 80% (NIH [16]) of human infections were associated with biofilms (Figure 1). While difficult to precisely evaluate, such estimates reveal the importance of studying biofilms in order to better understand their specific properties and fight them.

Development of simplified models started right after the demonstration of a link between sessile communities and human infections to study how bacteria, including pathogens, can form biofilms. Multiple *in vitro* models have emerged from scientists’ creativity, each of them especially adapted to observe biofilm formation of specific bacteria and within specific environments. The success of *in vitro* models but also their limitations, notably their failure to reproduce the host environment, led to a rapid development of multiple *in vivo* models ranging from surrogate non-mammalian models allowing high throughput analysis to more sophisticated models using rodents or higher animals such as dogs, pigs and monkeys. Interestingly, many of these models have been developed before clinicians and researchers realized that the modeled infection was indeed biofilm-related [18]. Importantly, because of justified ethical concerns, the use of mammalian *in vivo* models was early on restricted by legal regulations implying evaluation of scientific and medical benefits of the research but also taking into account animal welfare [19]. The protection of vertebrate animals therefore entails the evaluation of each project by ethics committees to confirm that they follow the “three R rule” edicted by Russel and Burch in 1959: Replace, Reduce and Refine [20]. This partially explains why *in vitro* and *in vivo* surrogate non-mammalian models are still heavily used and continue to reveal important insights about biofilm physiology and promising treatments for biofilm-related infections.

The aim of this review is to present the various aspects of the development of biofilm-related infection models ranging from simple *in vitro* to complex *in vivo* models. We will focus on discussing which experimental results have already or are about to reach clinical studies in humans. This review will also discuss specific future approaches that start to be used and should help to model better biofilm-related infections.

## 2. *In vitro* Biofilm Models

Simplified *in vitro* models have been instrumental in addressing basic questions about biofilm formation, physiology and architecture. They offer a number of advantages such as a low cost, easy set-up, and amenability to high throughput screens. They generally mimic hallmarks of biofilm biology like different gradients of nutrients, gases and metabolic products, as well as high cell densities or production and release of extracellular matrix. 

A complete and comprehensive list of *in vitro* biofilm models is provided in Table 1 and see [21,22] for further information. Briefly, the different *in vitro* biofilm models can be classified in three distinct groups:

(i) **Closed** or static models, in which there are limited nutrients and aeration. This includes some of the most popular and successful models, such as the colony biofilm model and microtiter plates [23,24]. In addition, these models enable direct rapid quantification of biofilm mass (*via* stains like crystal violet, safranin and congo red) or viable cells (XTT reduction assay).
(ii) **Open** or dynamic systems. The principle of these models is similar to continuous cultures, in which spent culture consisting of wastes, metabolic byproducts, dispersed and dead cells are constantly replaced by fresh medium. These methods generally allow the control of environmental parameters such as shear forces, and have been therefore extensively used to study the physical and chemical resistance of biofilms. However, they are in most instances less adapted to high throughput analysis and often demand specialized equipment and technical skills [22].
(iii) **Microcosms** are more sophisticated models that aim to closely mimic *in situ* conditions. They often include several bacterial species and use material from the studied environment, for instance, addition of hydroxyapatite and saliva to model dental biofilms [25], or covering abiotic surfaces with human cells in order to mimic an *in vivo* situation [26]. Theoretically, both open and closed systems could be turned into microcosms. Microcosms include more environmental parameters and take into consideration the complexity and heterogeneity of natural settings.

Although often regarded as over-simplistic, *in vitro* models have greatly contributed to today’s knowledge of biofilm physiology. They are still largely used to study the role of different genes involved in biofilm formation and regulation processes, as well as other applied purposes such as to screen libraries of antimicrobial agents. Nevertheless, these models ignore important parameters like environmental factors, or more specifically when characterizing pathogenic biofilms, host factors and other biotic signals.

**Table 1:** *In vitro* and *ex vivo* biofilm models.

Method	Characteristics	Advantages	Uses of model	References
1. Static systems				
Colony Biofilm	Colonies are grown over agar. Maintains basic biofilm characteristics like structured environment and chemical gradients.	Reproducible and simple. Amenable to high throughput screening.	Antibiotic susceptibility assessment Morphotypes observation essentially upon polysaccharides production	[381,382]
Microtiter plate	Commonly used. Bacteria attach to well surfaces.	Simple to run, Amenable to high throughput screening. Suited to molecular genetic tests.	Evaluation of biofilm formation of strains, biofilm antibiotic tolerance and resistance, efficiency of antibiofilm/antimicrobial products	[23,383]
Biofilm Ring Test	Immobilization of magnetic beads Results are automatized and analyzed by image analysis.	Allows for a rapid monitoring of biofilm formation. Possibility to assess early adhesion events. Quick and automatic analysis. Does not involve washing and staining procedures.	Evaluation of biofilm formation of strains	[384]
Calgary Biofilm Device	Based on 96-well microtiter plate assay. Includes a lid with 96 pegs on which biofilms develop.	Commercially available system. Pegs can be removed individually without opening the system, and hence avoiding contaminations. Consistent shear force across all pegs.	Biofilm antibiotic resistance and tolerance, efficiency of antibiofilm/antimicrobial products. Study biofilm development over time.	[385]
2. Open systems				
Kadouri system	Based on microtiter plate assay but with constant renewal of media. Minimum shear forces.	Formation of mature biofilm in microtiter plate wells, meaning a big amount of biomass that can be later used for microarrays and proteomics	Allows testing of multiple conditions and treatments.	[386]
Flow cell	Flat walled transparent chambers irrigated by culture media under the microscope. Costly and expertise is needed. System is automatized and available for image analysis.	Enables for non-destructive real-time biofilm observation (Allows single cell visualization). Excellent image quality	Evaluation of biofilm formation of strains, biofilm antibiotic tolerance, efficiency of antibiofilm/antimicrobial products.	[387]
CDC Biofilm reactors	Consists of eight polypropylene holders, accommodating 3 coupons each over which bacteria adhere, suspended from a lid surrounded by media	Commercially available system. Easy sampling at different time points Reliable system.	Evaluation of biofilm formation, biofilm antibiotic resistance and tolerance. Test disinfectant efficiencies. Study biofilm development over time.	[388]
Microfermentors	Chemostat-based Biofilms develop over a removable spatula composed of different materials	Large mass of biofilm produced Allows microscopic, genetic and biochemical analysis Different shear forces can be applied Can be easily turned into a microcosm	Easily converted into microcosms, by covering spatula with human cells. Evaluation of antibiotic effects and biofilm formation ability of strains.	[389]
Modified Robbins Device	Ports sit in a linear array along a rectangular channel. In each port, a plug can be inserted.	Sampling plugs can be removed and replaced aseptically	Used to mimic throat conditions and evaluate the efficiency of different products in rubber tracheo-oesophageal prostheses	[390]
Drip flow reactor	Individual channels are introduced into polycarbonate blocks within which microscope slides may be placed. Biofilms are grown in an angled way.	Low shear and high gas transfer. Allows for both solid-liquid and solid-air biofilm establishment.	Wound biofilm model. Tested for antimicrobial efficiency [391], bacteriophage reduction of biofilms and other antibiofilm strategies. Evaluation of disinfectant efficiencies. Study biofilm heterogeneity.	[392]
Microfluidic biochips	Biochip is embedded in an aluminium block in which temperature is controlled. Has contactless dielectric microsensors.	Non-invasive technique. Monitors dielectric changes of subcellular components within biofilm. Measures biofilm growth and development with sensitivity.	Useful to study population dynamics and quantitative cell analysis.	[393]
Constant depth film fermenter	Biofilms develop on polytetrafluoroethylene (PTFE) plugs. Biofilm growth and depth is limited. Excess biofilm is removed.	Excess biofilm is removed (imitating mechanical biofilm removal like tongue effect or toothbrush).	Specially suited to study oral biofilms. Tests of the effect of surface characteristic on biofilm formation. Antibiotic resistance tests.	[394,395]
Rotating Disc Reactor	Teflon rotor holding several (6 to 24) coupons over which biofilms will form. Rotor is embedded with a magnetic stir bar on the bottom and driven by a stirrer.	Liquid shear forces over the coupons can be varied.	Evaluate antimicrobial and antifouling treatments. Also used to study multispecies biofilms.	[396]
BioFlux Device	96 individual microfluidic channels fed with a pneumatic pump. Shear can be controlled individually in each channel.	Low cost in reagents and energy supply. High throughput analysis. Precise control of environmental conditions. Study of single cell behaviors within a community.	High throughput screening. Evaluation of biofilm formation of strains, biofilm antibiotic tolerance and resistance, efficiency of antibiofilm/antimicrobial products	[397]
Annular reactors	Based on two concentric cylinders; an outer static one which acts as the wall of the vessel and the inner rotating cylinder.	Shear forces can be controlled. Removable test coupons.	Evaluation of disinfection efficiencies. Study the effect of shear forces. Specially suited to study aquatic biofilms	[398]
Sorbarod devices (SBF)	Sorbarod filter plugs with a cellulose matrix perfused with media.	Easy set up. Substantial amounts of biomass. Growth rate control possible. Allows sampling of dispersed cells.	Used to evaluate long-time effects of antibiotics.	[399]
Perfused (membrane) biofilm fermenter	Cells are collected by pressure filtration in a cellulose acetate membrane. Filter is the inverted into the base of a modified fermentor Filter is the perfused with fresh medium Newly formed and loosely attached cells are eluted with spent medium.	Allows growth-rate control bacteria or yeast Adherent bacterial biomass is constant and proportional to the limiting nutrient concentrations	Used to evaluate antibiotic and fungicide efficiency	[400]
3. Microcosms				
Reconstituted Human Epithelia (RHE)	Biofilms form on top of human keratinocytes derived from buccal mucosa.	Takes into account some host factors, such as receptor specificity.	Human cells - bacteria biofilms interactions. Used to study oral biofilms.	[401]
Zürich Oral Biofilm-model	Biofilms form on hydroxyapatite disposed in 24-well microtiter plates	Can study population dynamics and antibiotic resistance and tolerance at the same time Semi high throughput	Used to study oral biofilms.	[402]
Zürich Burn Biofilm-model	Polymicrobial biofilms are grown on gauze discs of DermaPlast recovered by a protein pellicle disposed on 24-welled microtiter plates.	Allows the study of structure of polymicrobial biofilms. High repeatability.	Mimics biofilms development on burns. Suitable to assess antimicrobial efficiencies.	[403]
Endothelial Cells Under Flow model	Biofilms forms on human microvascular endothelial cells attached to microscope slide, perfused with media, under an inverted fluorescent microscope.	Has a continuous flow of nutrients. Biofilm development can be imaged and cells can be tracked. Takes into account shear forces in blood vessels.	Biofilm formation and dynamics on blood vessels and valves.	[404]
Airway Epithelial cell Model	Airway epithelial cells are disposed on collagen-coated membranes.	Allows formation of air-liquid biofilm formation.	Models chronic rhinosinusitis, cystic fibrosis and other biofilm-related pulmonary infections.	[405]
Multiple Sorbarod device (MSD)	Modified SBF system with five replicate plugs.	Allows for multiple replicates.	Used to replicate oral microcosms, perfused with saliva and multispecies biofilms	[406]
Microfluidic Co-culture model	Microfluidic channels covered with HeLa cells over which biofilms form.	Analysis of host-bacteria interactions. Real-time visualization of biofilm development.	Used to mimic gastrointestinal colonization. Human cells – bacteria biofilms interactions.	[407]
*4. Ex vivo*				
Root canal biofilms	Extracted tooth are embedded in silicone putty and irrigated.	Irrigation of dental surfaces. Allows imaging.	Remove dental biofilms and root canal infections	[408]
Cardiac valve *ex vivo* model	Use of excised porcine heart valve.	Study initial bacteria and the valve tissue interactions. Adapted to imaging (field emission scanning microscopy).	Evaluate progression of endocarditis	[409]
Candidiasis in vaginal mucosa	Rabbit vaginas are placed in 6-well tissue culture plates.	Optimal for microscopic evaluations (confocal and scanning).	Model of candidiasis	[27]
RWV Bioreactor	System able to grow 3D structures. Bubble-free aeration: Maintains cell polarity, differentiation and extracellular matrix production:	Circumvents conventional monolayers limitations. Minimizes mechanical cell damage. Microgravity conditions are maintained.	Has been used to model *P. aeruginosa* infection in lungs, *Salmonella* in gut and uropathogenic *E. coli*	[410,411,412]

## 3. *Ex vivo* Biofilm Models

Midway between *in vitro* and *in vivo* lie *ex vivo* models, in which tissues or organs are extracted from an organism (typically porcine or murine) and placed in an artificial environment for further analysis and experimentation. Often neglected, they allow for more controlled experimental conditions than *in vivo* models and can provide an alternative to living organisms in order to perform otherwise ethically questionable measurements and experiments. They can be particularly useful to image or analyze the progression of bacterial colonization of a given organ or tissue, such as tracheal epithelium, vaginal mucosa, kidneys or teeth [27,28,29,30]. They can also be used to assess different time-windows for effective treatment of biofilm infections [31]. Details of different *ex vivo* models are described in Table 1. 

## 4. Non-Mammalian *in vivo* Models

Infection and pathogenesis is a continuous interplay between the host and microbes and between microbes themselves. These interactions can influence and determine the fate of infection and they are complex and dynamic, which makes it difficult to study them in a relevant manner in *in vitro* models [32]. As *in vitro* models offer a simplified vision of the environment, it is important to use adequate *in vivo* models to validate *in vitro* results as a first step to test hypothesis that could be later translated into higher organisms or clinical settings. 

**Figure 2:**
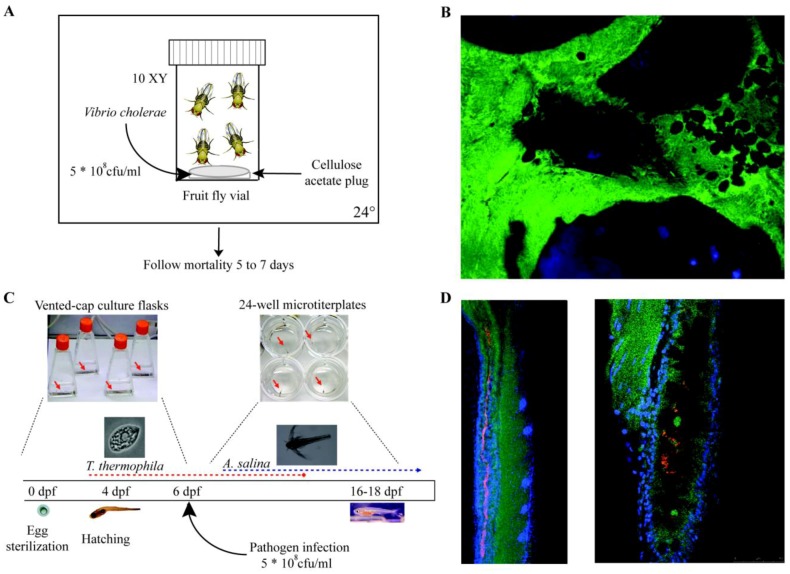
Non-mammalian *in vivo* models. **A**. Experimental settings. *Drosophila melanogaster*. Ten male fruit flies are selected and introduced in standard fly vials. A dilution of a *Vibrio cholerae* overnight culture to 5 * 10^8^ CFU/mL is used to impregnate a 0.5-inch cellulose acetate plug placed at the bottom of each vial. Then, the vials are kept at 24 °C with appropriate light-dark cycles. Fruit fly survival is monitored twice a day for 5 to 7 days. **B**. Confocal microscopy image of *D. melanogaster* rectum papillae (oval structures) colonized by a *V. cholerae* (gfp-tagged, green) biofilm. Cell nuclei are stained in blue (DAPI staining). Images Credit: A. Purdy and P.I. Watnick Division of Infectious Diseases, Children’s Hospital, Boston, USA. Adapted from [38]. **C.** Experimental settings. Axenic zebrafish infection. After fertilization, eggs are immediately sterilized and kept in vented cap cell culture flasks in autoclaved mineral water at 28 °C. Starting at 4 dpf (days after fertilization), larvae are fed every 2 days with axenic *Tetrahymena thermophila* until day 15. For longer experiments, in addition to *T. thermophila*, larvae were fed axenic *Artemia salina* from 10 dpf onwards. Zebrafish larvae are infected 6 days after fertilization with 5 * 10^8^ CFU/mL of pathogen. Mortality can be easily followed on daily basis. Adapted from [41]. **D.** Confocal fluorescence pictures of larval intestine infected by the pathogen *E. ictaluri* (detected by immunofluorescence, red) 1 day after infection. Zebrafish cell nuclei are shown in blue (DAPI staining) and actin in green. Images Credit: J.P. Levraud and M. Frétaud, Institut Pasteur, Paris, France.

In the past ten years, in order to overcome the practical difficulty associated with the use of mammalian models, non-mammalian models traditionally used to study development like the fruit fly, *Drosophila melanogaster* or the zebrafish, *Danio rerio*, have been adapted to study host-microbe interactions and immune system responses, notably related to colonization of the gut by biofilms [33]. Increasing awareness of biofilm-related infections has prompted these and other models discussed in Table 2 and Figure 2, to study tissue colonization, biofilm formation and the onset of pathogenesis. Many different models have been proposed, ranging from simple plant models such as *Arabidopsis thaliana* and *Lemna minor,* which were successfully used to correlate virulence and biofilm formation in pathogenic *S. aureus* and *P. aeruginosa* [34] to more complex invertebrates like *Caenorhabditis elegans* [35], *D. melanogaster* (Figure 2) [36,37,38] or the vertebrate zebrafish [39,40,41] (Table 2 and Figure 2).

**Table 2:** Non-mammalian *in vivo* models.

Organism	Size	Generation time	Temp. (°C)	Immune system	Follow-up of host infection	Relevant Model	Human Pathogens studied	References
*Tetrahymena pyriformis*	20 × 40 μm	7 hours	22–26	Unknown	Real-time through bacterial fluorescent markers	Biofilm grazing, Virulence and toxicity	*Klebsiella pneumoniae, Legionella pneumophila, Vibrio cholerae*	[413]
*Acanthamoeba sp.*	15 to 35 μm	6–10 hours	19–25	Macrophage analog	--	Biofilm grazing, Phagocytosis, intracellular survival	*L. pneumophila, Cryptococcus neoformans, Candida albicans,* Methicillin-resistant *Staphylococcus aureus* (MRSA), *V. cholerae*	[414]
*Dictyostellium discoideum* (Slime mould)	10–20 μm	4–12 hours	19–25	Macrophage analog	Real-time through bacterial fluorescent markers	Biofilm grazing, Phagocytosis, intracellular survival	*Pseudomonas aeruginosa, L. pneumophila, Listeria monocytogenes* (intracellular pathogens)	[415]
*Lemna minor* (Duckweed)	2–5 mm × 1.5–3.5 mm	1 week	28	Unknown	✗	Biofilm formation and virulence	*S. aureus, P. aeruginosa, Salmonella spp., Shigella spp., Yersinia spp.*	[34]
*Medicago sativa* (Wounded alfalfa)	Seedlings	3 months	30	Unknown	✗	Chronic bacterial lung infections,	*P. aeruginosa, Burkholdheria cepacia*	[416]
*Arabidopsis thaliana* (Thale cress)	1 to 20–25 cm	3 weeks	20–25	Analog pathwaysto MAPK	✗	Biofilm formation and virulence	*Pseudomonas* spp., *S. aureus*	[417]
*Hirudo sp* (Leach)	15–40 mm (adult)	--	10–35	Unknown	--	Biofilm competition and gut colonization	*Aeromonas* spp.	[418]
*Panagrellus redivivus* (Sour paste nematode)	1 mm × 50 μm	3–5 days	37	Innate immunity (Toll-like receptor, MAPK)	--	Biofilm formation, virulence, gut colonization	*P. aeruginosa, Salmonella enterica,* and *S. aureus*	[419]
*Caenorhabditis elegans* (Round worm)	1 mm ×100 μm	4–7 days	22–27	Innate immunity (Toll-like receptor, MAPK)	Real-time through bacterial fluorescent markers	Biofilm formation, virulence, gut colonization	*Microbacterium nematophilum, Escherichia coli, Shigella flexneri, V. cholerae, Shewanella* spp. *Listeria* spp., *S. aureus, Streptococcus sp.*	[420]
*Galleria mellonella* (Wax moth caterpillar)	3 cm in length	--	30	Innate immunity (Toll-like receptor, MAPK, NFκB)	--	Biofilm formation and virulence	*Pseudomonas* spp., *Proteus mirabilis, E. coli, Bacillus cereus, Bacillus thuringiensis, C. albicans, C. neoformans*	[421]
*Drosophila melanogaster* (Fruit fly)	3 mm	10 days	12–30	Innate immunity (Toll-like receptor, Imd, MAPK, NFκB)	Real-time through fluorescent markers, LacZ fusions available	Biofilm formation, virulence, gut colonization	*Wolbachia* spp., *Serratia marcescens, Erwinia* spp., *Pseudomonas entomophila, C. neoformans, Francisella novicida, L. monocytogenes, V. cholerae, C. albicans*	[422]
*Danio rerio* (Zebrafish)	3–5 mm (larvae) 6–6.5 cm (adult)	3–4 months	23–28	Adaptive and innate	Real-time through fluorescent markers both on host and bacteria	Biofilm formation, virulence, gut colonization	*Mycobacterium marinum, Oodinium, Microsporidia, E. coli, Pseudomonas* spp., *Salmonella* spp. *Vibrio* spp.	[423]

^1^ As insects, they could also be used to model gut colonization and commensal-pathogen interaction. However, to the best of our knowledge, it has not yet been used with this objective. ✗: not possible; --: not described.

Non-mammalian models share advantages such as a rapid development leading to short generation time and are generally cheap to raise and perform experiments. In addition, most of these model organisms have their genome already sequenced and can be genetically manipulated, thus, offering the possibility to do genetics both on the bacteria and the host. In addition, their small size enables to maintain most of them in microtiter plates, which is useful to perform high throughput studies, some of which in an automated version [42]. This has already allowed successful screening for virulence genes [39,43,44,45], colonization and biofilm formation factors [34,46,47] or chemical libraries for antimicrobial compounds [48]. 

Their reduced complexity for some pivotal systems linked to infection has actually been an advantage. For example, despite the simplicity of their immune system, these surrogate non-mammalian organisms have been useful to identify evolutionarily conserved host defense mechanisms and have shed light into universal immune mechanisms [49]. Additionally, the facility of generating axenic or gnotobiotic animal models has allowed simplifying host/pathogens and flora interactions studies. Recent research using an axenic zebrafish model has proven that increased biofilm forming ability of commensal bacteria can rescue larvae from *Edwardsiella ictaluri*-induced death [41].

Nevertheless, these models reached their limits when studying complex immune responses in relation to biofilm infection or using pathogens whose optimal growth temperature and expression of virulence factors are beyond the animal growth temperature. Moreover, due to the short lifespan and duration of experiments, these models seem unsuited to study chronic infections typically caused by biofilms.

## 5. Tissue-Associated Biofilm Models

While important information has been generated using *in vitro* and invertebrate *in vivo* models, the use of mammalian models that are closer to humans is required before considering going from bench to bedside. Therefore, many efforts have been made to closely mimic in higher organisms the occurrence of biofilm-related infections that allowed us to address diagnostic or therapeutic challenges (see Table 3). 

**Table 3:** *In vivo* models of biofilm-related infections.

Type of biofilm-related infection	Type of model	Animal	Microorganisms	Direct biofilm /chronic infection	Technical details	References
Tissue-related infections						
CF lung infections	Agar-bead based infection model	Rats, mice, cats, guinea pigs and monkeys	*P.a, S.a, H.i* and *B. cenocepacia*	B	Intratracheal route of infection	[54,55,56,57,58,59]
	Seaweed alginate microsphere infection	Rats, mice, guinea pigs	*P.a*	B	Intratracheal route of infection	[62,63,64]
	Agar-bead based model	Mice	*P.a*	B	Intravenous injections	[66,67,424,425]
	CF model (CFTR-/- mice)	Mice, pigs, ferrets	*P.a, S.a and B. cepacia*	B	Agar-bead based intranasal route of infection	[68,70,74,426]
COPD associated infections	COPD/emphysema	Mice	*H.i*	B	Intranasal route of infection	[76]
Diffuse panbronchiolitis	Chronic diffuse panbronchiolitis	Mice	*P.a*	CI	Piece of intravenous catheter coated with *P. aeruginosa*	[80,81,82]
Urinary tract infections	Murine cystitis model	Mice	*E.c, K.p*	B	Transurethral catheter for inoculating bacteria in bladder	[89,91]
	Rat model of chronic cystitis	Rats	*E.c*	CI	Transurethral catheter for inoculating bacteria in bladder	[86]
Chronic bacterial prostatitis	Experimental model of chronic prostatitis	Rats	*P.m, E.c*	CI	Prostatic urethral injections	[115,117,118]
Urinary Stones or Struvites	Rat model of urolithiasis	Rats	*P.m, U. urealyticum*	B	Foreign body like zinc disc or chalk in bladder	[127,129,130,427]
Pyelonephritis	Urinary stone genesis model	Rats	*P.m*	B	Zinc discs in bladder	[127]
Intestinal Infections	Intestinal colonization model	Mice	*C. rodentium*		Oral dosing of bacteria	[141,142]
	Streptomycin-treated mouse model	Mice	*E.c, Salmonella*	B	Oral dosing of bacteria	[138,140]
Gall Bladder Infections	Chronic infection model	Mice	*Salmonella*	CI	Oral infection	[145,146,147]
Chronic wounds infection	Needle scratch model, Skin abrasion	Mice	MRSA	B	Scratch with 28 gauge needle on skin to damage epidermis	[152]
	Wound infection model	Mice	MSSA	B	Full-thickness wound is established through the panniculus carnosus on the back of animals	[158]
	Excisional wound model	Mice	*S.a*	B	Cuts made on the back of mice	[156]
	Ischemic wound model	Rats	*P.a*	B	Pressure-induced wounds	[159]
	Cutaneous wound healing model	Rabbits	*P.a*	B	Circular punch-wounds in ear	[428]
	Cutaneous porcine wound model	Pigs	*S.a*	B	Partial thickness wounds on paravertebral area using a modified electrokeratome	[157]
	Diabetic foot wound model	Mice	*E.c, B. fragilis* and *C. perfringens*	B	Leptin-receptor deficient mice injected in inner thigh	[161]
Infective endocarditis	Catheter-induced IE	Rabbits	*S.a*	B	High inoculum of bacteria injected intravenously	[164,166]
	Low-Grade bacteremia model of IE	Rats	*L. lactis*	B	Low-grade inoculum of bacteria injected intravenously	[169]
Chronic otitis media	COM with effusion	Gerbils	*H.i, S. pneumoniae*	B	Injected percutaneously into the superior posterior chamber of the left middle ear	[429]
	Chinchilla Model of COM	Chinchillas	*H.i, P.a, group A Streptococcus*	B	Bacteria is injected bilaterally via a transbullar approach	[183,186]
	Nonhuman primate model of COM	Cynomolugus macaques	*P.a*	B	Perforation of the tympanic membrane and inoculation of the middle ear	[190]
	COM	Rats	*P.a*	B	Intranasal inoculation using teflon cannula	[191]
	COM	Mice	*S. pneumoniae*	CI	Spontaneous OM development in plasminogen deficient mice	[192]
Chronic rhinosinusitis	Chronic rhinosinusitis	Rabbits	*S. pneumoniae*	CI	Hole drilled into the dorsum of nose, cotton wool inserted and inoculated with 10.8 bacteria	[195]
	Chronic rhinosinusitis	Mice	*L. sakei, C. tuberculostearicum*	CI	Intranasal inoculation	[197]
	Experimental rhinosinusitis biofilm model	Sheep	*S.a*	B	Ostium occluded and bacteria instilled	[198]
Dental caries	Experimental caries	Hamsters	*S. mutans*	CI	Oral inoculation	[206]
	Model of Cystic Fibrosis	Mice	*S. mutans*	B	Swabbing the oral cavity of CFTR knock out mice	[205]
	Model of periodontal disease	Rats	*P. gingivalis*	B	Topical administration of bacteria	[207]
Periodontitis	Oral infection model	Mice	*T. denticola, P. gingivalis*	CI	Oral inoculation by gavage	[207,223]
	Experimental periodontitis	Mice	*F. nucleatum, P. gingivalis, T. forsythia*	B	Oral gavage using a feeding needle	[222,224]
	Experimental periodontitis	Rabbits	*P. gingivalis*	B	Oral inoculation	[430,431]
Osteomyelitis	Chronic osteomyelitis	Rabbits	*S.a*	CI	Injection in tibial metaphysis into the intramedullar cavity	[229]
	Osteomyelitis model of biofilm	Rabbits	*S.a*	B	Injection in tibial metaphysis into the intramedullar cavity	[231,232]
	Experimental chronic osteomyelitis	Rats	*S.a*	CI	Hole is drilled into the medullar cavity, bacteria are injected into the bone	[233]
	Experimental model of osteomyelitis	Mice	*S.a*	CI	Bioluminescent strain of *S. aureus* is inoculated into the femurs of mice	[234]
Device related-infections						
Vascular Catheter	CVC	Rats	*S.e, S.a, C.a*	B	Catheter tip in superior vena cava through jugular vein, tunneled subcutaneously and exits on the back. Use of restraint jacket	[239,245,432]
	CVC	Rabbits	*S.e, S.a*	B	Catheter tip in superior vena cava through jugular vein, tunneled subcutaneously and exits on the back. Use of restraint jacket	[247,252,253]
	Totally implantable venous access port	Rats	*S.a, S.e, P.a, E.c*	B	Catheter tip in superior vena cava through jugular vein, tunneled subcutaneously and connected to a subcutaneous port	[256]
Urinary tract catheters	Bladder glass bead (surgical)	Rats	*E.c*	B	Bead colonized by *E. coli* biofilm surgically inserted in the bladder. Urethra clamped 1h/day to reproduce vesico-ureteral reflux	[260]
	Bladder pieces of catheter (surgical)	Rats	*P.a*	B	Surgical insertion of pieces of urinary catheter. Bacterial inoculation is made inside the bladder, after catheter insertion	[261]
		Mice	*E.c*	B		[262]
	Bladder pieces of catheter (non surgical)	Rabbits	*P.a, P.m*	B	Use of urethral catheter and/or metal stylet in order to transurethrally insert pieces of catheter inside the animal bladder	[263,265]
		Rats	*P.a*	B		[433]
		Mice	*P.a, P.m, E.c, E. faecalis*	B		[264,266]
	Externalized urethral catheter	Rabbits	*E.c*	B	Urethral catheter inserted and connected to a urine collector *via* a closed system in order to mimic an externalized system	[269,270,271]
		Sheep		B		[272]
Orthopedic implants	Foreign-body in tibia	Rabbits	*S.a*	B	Silicone rubber catheter inserted into the tibia and associated with sclerosing agent to induce aseptic necrosis	[277,434]
	Foreign-body in tibia	Rabbits	*S.a*	B	Titanium cylinder or bone cement inserted into the tibia. Bacterial inoculation up to 4 months after foreign-body placement	[279,435]
	Electrode inserted in tibia	Rabbits	*S.e*	B	*S. epidermidis* is injected inside tibia through a hole. A stainless steel electrode is inserted inside medullar cavity + bone cement	[436]
	Devascularized bone and metal screws	Rabbits		B	A piece of diaphyseal radial bone is removed. Then, this devascularized bone is replaced inside the wound using metal screws	[280]
	Spinal device model	Rabbits	*S.a*	B	Partial laminectomy followed by a wire implantation of the transverse processes of different vertebra (T13, L3, L6)	[437]
	Titanium wire inside tibia	Rats	*S.a*	B	Insertion in the medullar canal of a long titanium wire. Before the insertion of the foreign-body, *S. aureus* inoculum is injected	[282,283]
	Stainless steel pin inserted through tibia	Mice	*S.a*	B	Stainless steel pin incubated 20 minutes with bioluminescent *S. aureus* and then inserted transcortically through mice tibia	[281,438]
	Bone cement in tibia	Dogs	*S.a*	B	Removal of a cortical part of tibial metaphysis. Then, polymethylmethacrylate cement and *S. aureus* are inserted in bone pocket	[284]
	Cylindrical device in femoral canal	Dogs	*S.e, S.a, E.c*	B	Cylindrical device (made of stainless steel, cobalt chromium, polyethylene or polymethylmetacrylate) inserted inside femoral canal	[278]
	Intramedullar nail	Dogs	*E.c, P.a*	B	Mid-diaphyseal osteotomy and internal fixation with an intramedullar nail inoculated, before fixation, with *E. coli* and *P. aeruginosa*	[439]
	Fracture fixation stainless steel plates	Sheep	*S.a*	B	Membranes colonized by *S. aureus* biofilms are positioned on stainless steel plates and drilled on a cortical surface of sheep tibia	[440]
Prosthetic joints	Hemiarthroplasty and bone cement	Rabbits	*E.c*	B	Knee hemiarthroplasty and acrylic bone cement followed by intraarticular *E. coli* injection	[287]
	Total knee replacement	Rabbits	*S.a*	B		[288]
	Hip stainless steel prosthesis	Rabbits	*S.a*	B		[441]
	Silicone-elastomer implant	Rabbits	*S.a*	B	Partial knees arthroplasty using silicone-elastomer implants. At the end of surgical procedure, *S. aureus* is injected into the joint	[286]
	Pin inside femur with the tip in the joint	Mice	*S.a*	B	Stainless steel pin is inserted inside the femoral canal and the distal end of the pin protrudes inside the joint space	[289]
Endotracheal tubes	Ventilated sheep	Sheep	Oral flora	B	Animals are intubated and ventilated for 24 to 96 hours before being sacrificed for endotracheal tubes analysis	[293,297]
	Ventilated pig with induced pneumonia	Pigs	*S.a*	B		[294]
	Ventilated dog	Dogs	*P.a*	B		[295]
Vascular grafts	Infrarenal aortic vascular graft	Dogs	*S.e*	B	Implantation of dacron prosthesis colonized by *S. epidermidis* on the infrarenal aorta of a dog leading to a prosthetic graft infection	[300,442,443]
	Infrarenal aortic vascular graft	Pigs	*S.a*	B	Surgical implantation in the infrarenal abdominal aorta of vascular grafts colonized by *S. aureus*	[303]
Tissue fillers	Breast implants	Pigs	*S.e*	B	Each pig received up to 6 miniature silicone gel-filled implants into submammary pockets + inoculated with *S. epidermidis*	[306,307]
	Breast implants	Rats	*S.a*	B		[444]
Contact lenses	Damaged cornea and contact lenses	Mice	*Fusarium spp.*	B	*Fusarium* spp. are grown as biofilm on silicone hydrogel contact lenses and mouse cornea are damaged by scratches or abrasion	[311]
Dental implants	Titanium screw into hard palate	Rats	*A. actinomycetemcomitans*	B	Biofilm-inoculated titanium implants transmucosally placed into rat hard palate	[315]
	Intrabuccal splints and disks	Humans	Oral flora	B	A removable fixation system applied on the mandibular region, inside the mouth of healthy volunteers. On these splints, disks made of various surfaces can be placed to study biofilm formation	[316]
Subcutaneous models	Tissue cage	Mice, rats, hamsters, guinea pigs, ponies	*S.a, S.e, A. radicidentis*	B	Rigid tubes, mostly made of teflon and perforated with holes, sealed at each end, possibly filled with glass bead and usually inserted in the flank of animal. Tissue cage fluid can be collected by percutaneous aspiration	For review: [22]
	Vascular catheter	Mice, rats, rabbits	*S.e, S.a, E.c*	B	Insertion of a 1-cm segment of vascular catheter in a subcutaneous space. At the end of the experiment, mice are euthanized, catheter segment is removed, vortexed in order to recover the biofilm	For review: [22,243]
	Cement disks	Rabbits	*E.c*	B	Acrylic bone cements shaped like disks and colonized by *E. coli* biofilm are surgically inserted into subcutaneous pockets on the back	[329]
	Pacing device	Rabbits	*S.e, S. capitis, E. c and A. baumannii*	B	Pacing device is inserted in subcutaneous pockets on the back of the animals. Bacterial contamination is made inside the pocket, at the end of the experiment	[330]
	Fabric to mimic cardiac valves	Guinea pigs, mice, rabbits	*S.e, P.a*	B	Various types of implants impregnated with antibiotics or not are incubated with a bacterial solution in order to allow biofilm formation. Afterwards, these devices are inserted into subcutaneous pockets	[331,332,334]
	Vascular grafts	Hamsters, mice, rats	*S.e*	B	Gore-tex implants colonized by *S. epidermidis* biofilm are inserted into subcutaneous pockets	[335,336,445]
	Polyethylene disks	Mice, rabbits	*E.c, P.a*	B	Subcutaneous implantation of polyethylene disks	[338]
	Beads	Rats	*S.a*	B	Polymethylmethacrylate beads loaded or not with various compounds are inserted in subcutaneous space	[339]
	Surgical mesh	Mice	*S.a*	B	Insertion of resorbable or non resorbable surgical meshes colonized by *S. aureus* biofilm inside subcutaneous pockets	[340,341]

**NOTE:** B: biofilm, *C.a: Candida albicans*, CVC: Central venous catheter, CI: chronic infection, COPD: chronic obstructive pulmonary disease, COM: chronic otitis media, CF: cystic fibrosis, *E.c: Escherichia coli, H.i: Haemophilus influenzae*, IE: Infective endocarditis, *K.p: Klebsiella pneumoniae*, MRSA: methicillin-resistant *S. aureus*, MSSA: methicillin-susceptible *S. aureus*, *P.m: Proteus mirabilis, P.a: Pseudomonas aeruginosa, S.a: Staphylococcus aureus, S.e: Staphylococcus epidermidis*.

The role of biofilm in the pathophysiology of tissue-associated infection has been increasingly recognized in the past decades (Figure 1). Biofilm may develop after colonization of sterile or non-sterile tissue or mucosa by microorganisms or may take advantage of the prior alteration of resident commensal flora by antibiotics.

### 5.1. Lung Infections

#### 5.1.1. Cystic Fibrosis (CF) Related Lung Infections

CF disease is the result of a single gene mutation in cystic fibrosis transmembrane conductance regulator (CFTR) leading to a multitude of medical problems amongst which are pancreatic failures, alteration of mucosal secretions and of epithelial innate immune function in the lungs. However, the most challenging aspect of the disease is pulmonary infection that leads to 80–95% of respiratory failure by chronic bacterial infection and airway inflammation [50,51,52]. While previously suggested by Lam *et al.* and Hoiby *et al.*, who showed the presence of matrix-embedded microcolonies of *P. aeruginosa* in chronically infected lungs [12,14], confirmation of the role of biofilms was provided by Singh *et al.*, through identification of a specific quorum sensing signal signature [53].

Animal models used to mimic CF infected lungs can be broadly divided based on the route of infection chosen: intratracheal or intravenous.

- *Intratracheal route:* Cash *et al.*, were the first to describe a rat model to establish *P. aeruginosa* chronic lung infection, lasting up to 1 month, by immobilizing bacteria in agar beads [54]. This model was later adapted to mice, guinea pigs, cats, monkeys and to other pathogenic agents like *S. aureus*, *Burkholderia cenocepacia* and *Haemophilus influenzae* [54,55,56,57,58,59]. This model depicts human clinical pathologies such as bacterial persistence and airway inflammation [60]. Amongst very recent uses, it successfully evaluated the efficacy of liposomal bismuth-ethanediol loaded tobramycin against *P. aeruginosa* infection in rat lungs [61]. Another rat model developed by Pedersen *et al.,* used bacteria embedded in seaweed alginate microspheres [62]. Amongst other advances, this model showed the role of Psl and Pel polysaccharides as a scaffold of biofilms formed by mucoid *P. aeruginosa* phenotype biofilms in mice [63]. In addition, to study pharmacokinetics/dynamics of colistin and imipenem, a neutropenic mouse model of lung biofilm infection was developed [64].
- *Intravenous route*: Developed by Sawai *et al.* [65], this model also makes use of pathogens embedded in agar beads, which are afterwards injected intravenously to mice. Efficiency of several antimicrobials, such as linezolid, quinolones and carbapenems was assessed using this model [66,67].

A relevant infection model of CF should reflect human lung infection characteristics such as airway inflammation and spontaneous bacterial infection progressing to chronic stage with characteristic biofilm formation. An attempt to achieve this was made in 1992 with a CFTR knock out mouse [68]. Though mice did not show spontaneous lung disease, airways inflammation and chronic infection were established, and this model was successfully used to model chronic *P. aeruginosa* lung infections using agar beads [69] and to evaluate the efficacy of azithromycin against *P. aeruginosa* biofilms [70]. The CFTR knockout mice provided important information on the molecular mechanisms explaining the efficacy of azithromycin in clinical trials [71]. Recently, this model was also used to mimic lung co-infections by *P. aeruginosa* and *B. cenocepacia* [72]. CFTR knockouts were later translated into pigs (ΔF508/ΔF508) and ferrets. They were used to study *S. aureus* pathogenesis in CF [73] and general CF pathology [74], respectively.

#### 5.1.2. Other Lung Conditions

*Chronic obstructive pulmonary disease (COPD) related lung infections*: COPD is a progressive lung disease characterized by emphysema, chronic bronchitis and bronchiectasis. The etiological agents of COPD-related airways infection include non-typeable *H. influenzae* (NTHi), *Streptococcus pneumoniae*, *Moraxella catarrhalis* and *P. aeruginosa* [75]. So far, only one mouse model of COPD-NTHi lung infection has been reported and allowed to demonstrate the presence of persistent multicellular bacterial communities in elastase-damaged lungs and the role of CD54 in NTHi clearance [76].

*Diffuse panbronchiolitis (DPB)*: DPB is characterized by thickening of bronchiolar walls and is commonly associated with accumulation of lymphocytes, plasma cell and histiocytes [77]. There is large evidence involving bacterial biofilms in DPB [78,79]. To mimic chronic *P. aeruginosa* DPB infection in the respiratory tract, a murine model was developed in which a small piece of intravenous catheter (tube) precoated with *P. aeruginosa* was inserted into the mouse trachea [80]. This model was used to study the efficacy of macrolides against chronic *in vivo* *P. aeruginosa* infections and the role of inflammatory cytokines and dendritic cells in pathogenesis of DPB [81,82].

### 5.2. Urinary Tract Infection (UTI)

Urinary tract infections, which include infection of bladder (cystitis) and kidneys (pyelonephritis associated with or without kidney stones) primarily affect women and account for nearly 13 million annual doctor’s visits in the United States alone [83]. Recurrence of UTI is a major concern with 20–30% incidences among adult women. However, elderly and prepubertal children are also susceptible to recurrent and chronic cystitis [84]. Etiology of UTI is dependent on host genetics, biological and behavioral factors and has been largely associated with presence of *Escherichia coli* (*E. coli*) (80% cases) and to a lesser extent to *Staphylococcus saprophyticus* (10–15%) followed by *Klebsiella, Enterobacter, Proteus* and *Enterococcus* species [83,85].

#### 5.2.1. Cystitis

Bacterial colonization of the bladder results in a mucosal inflammatory response called cystitis. Uropathogenic *E. coli* (UPEC) are the most widely studied bacteria [83]. Except for few studies using rats where preventive effect of fosfomycin trometamol (FOF) was evaluated against chronic cystitis [86], most information on UTI originated from mouse models. In addition to genome availability and repertoire of knockout mutants, mice are naturally susceptible to UPEC strains and experimental infection closely resembles human disease [83,87,88]. Mouse model of cystitis was efficiently used to demonstrate that uropathogenic strains of *E. coli* and *K. pneumoniae* exist in biofilm-like large aggregates of bacteria (pods or intracellular bacterial communities, IBC) in the bladder epithelial cells suggesting one possible mechanism of recurrent cystitis [89,90]. These pod-like structures were later shown to exist in human bladder during UTI [88]. Murine pod like structures exhibited resistance to antibiotics and host system [90,91] and were protected against trimethoprim sulfamethoxazole [92]. This model has been successfully used to study the molecular mechanisms involved in IBC formation and virulence of UPEC such as role two-component system QseBC [93], importance of peptide transport, TCA cycle and gluconeogenesis but not glycolysis [94]. Importantly, this model also highlighted the role of type 1 pilus and its associated tip adhesin, FimH, in IBC formation. Type 1 fimbriae were shown to mediate adherence and invasion of urothelial cells [95] *via* FimH affinity for mannosylated urothelium proteins such as uroplakin [95,96,97]. Consistent with the instrumental role of FimH in UPEC bladder colonization, vaccines against FimH as well as FimH specific inhibitors were later shown to be protective or efficient against established infection in murine and/or primate models of acute cystitis [92,98]. These anti-FimH strategies are very promising and clinical studies proving their efficacies are now expected [92,99]. Other prophylactic or curative strategies based on bacterial interference and immune system stimulation with non-adherent asymptomatic bacteriuria strain 83972 were successfully assessed using UTI mouse models and validated in patients [100,101]. 

Murine studies have also been extensively carried out to confirm the role of innate immune responses in disease outcome and recurrence and highlighted the importance of TLR4 signaling [83,102,103]. Furthermore, CD8+ T cells were reported to play a role in adaptive response to UPEC bladder infections [104]. Murine UTI model led to identification of biomarkers associated with chronic cystitis including elevated serum IL-5 and urine IL-6, G-CSF (Csf2) and KC (CXCL1) [105,106] and more recently the autophagy gene *Atg16L1* was shown to play an important role in pathogenesis of UTI [107]. 

#### 5.2.2. Chronic Bacterial Prostatitis (CBP)

CBP is a persistent inflammation of the prostrate glands due to bacterial invasion. CBP may account for 5–10% of total prostatitis pathology [108]. First evidence of biofilms from biopsied prostatic tissues confirmed the presence of sparse microcolonies of *S. aureus* in the prostatic duct walls [109]. The major causative agents are from the *Enterobacteriaceae* family, like *E. coli* but also *Enterococcus faecalis,* Pseudomonads, *Staphylococcus* species and gonococcal organisms [108,110,111,112]. The capacity of these bacteria to form biofilms containing antibiotic persistent bacteria explains recurrent CBP [111,113,114]. The rat model has been extensively used to demonstrate the importance of virulence factors or quorum sensing systems in CBP [115,116,117]. This model was also used to evaluate therapeutic strategies against *E. coli-*induced CBP using catechin, ciprofloxacin, or cranberry [118,119] as well as the potential of selenium in combination with ciprofloxacin [115].

#### 5.2.3. Other UTI

*Infectious urinary stones/calculi or Struvites:* Persistent infections caused by urease producing bacteria may lead to rapidly growing infectious stones within 4–6 weeks [120,121]. Initial clues of biofilm involvement in infectious stones date back to 1971 when Nemoy *et al.*, showed the presence of antibiotic resistant bacteria embedded in stone resulted in recurrent urinary infections [122]. The first hypothesis that such antibiotic resistance was due to bacteria growing within glycocalyx matrix of biofilms [10] was later confirmed by scanning electron microscopy and transmission electron microscopy [123]. The most common urease producers involved in infectious stones are: *Proteus* spp., *Ureaplasma* spp., *Pseudomonas* spp., *Klebsiella* spp., and *Staphylococcus* species [124]. Moreover, *Oxalobacter formigenes* and *Lactobacillus* were also suggested to cause nephrolithiasis [125,126]. Rat is the most widely utilized animal to develop experimental infectious stones and most of the studies were performed with *Proteus mirabilis* and *Ureaplasma urealyticum* [127,128,129]. Using a foreign body such as zinc or chalk to develop bladder stones helped understanding the kinetics of stone formation and the role of *P. mirabilis* biofilms in this process [129,130]. This type of infectious urolithiasis rat model was also used to study therapeutic strategies against polymicrobial (*P. mirabilis, P. aeruginosa* and *E. faecalis*) stone infection [128].

*Pyelonephritis:* Once bacteria enter the kidney, they adhere to the urothelium and papillae to form biofilm-like structures [131]. While numerous models of acute pyelonephritis have been developed, the presence of biofilms was demonstrated solely for *P. mirabilis* biofilms on the rat urothelium [127].

### 5.3. Digestive Infections

#### 5.3.1. Intestinal Infections

The human gastrointestinal tract is covered by a biofilm of commensal bacteria that form independent communities depending on the colonized niches [132,133]. Similarly, biofilms were shown to colonize the mucus layers of large intestine of other animals such as baboons, rats and mice [134,135]. The gut biofilm is shaped by different factors, both environmental and specific to the host [136]. Alterations of the flora equilibrium might lead directly to pathology, altered physiological and immunological states due to changes in the functional microbial core. Gut diseases can be separated in two groups: acute diseases such as diarrhea that are generally due to a pathogenic bacteria displacing the commensal flora and impacting directly on intestinal epithelial cells, and chronic diseases such as inflammatory bowel disease, commonly named IBD, a group of inflammatory conditions of the colon and the small intestine that can evolve to colorectal cancer and that have been partly linked with a microbial imbalance of the gut commensal flora (dysbiosis) [137]. The streptomycin-treated mouse model of intestinal colonization developed long ago [138,139] has been extensively used to study both virulence factors and hosts components involved in intestinal colonization (see for example [140]). In addition, infectious colitis models of gnotobiotic mice or conventional mice colonized by their natural pathogens such as *Citrobacter rodentium* have been used [141,142].

#### 5.3.2. Gall Bladder Infections

*Salmonella enterica* serovar Typhi is the causal organism of typhoid fever and is amongst the best-studied gut bacteria. Inability of antibiotics to resolve the *Salmonella* Typhi colonization of the gall bladder indicated the role biofilm in this chronic disease [143,144]. A mouse model of chronic *Salmonella* infections was developed by Sukupolvi S *et al.* [145] and was later used to analyze chronic infections that persisted for 1 year following oral infection [146]. Crawford RW *et al.*, (2010) recently developed another mouse model of enhanced *S.* Typhimurium colonization of gall bladder tissue, which allowed visualization through electron microscopy of a dense biofilm covering more than 50% of gallstone surface [147]. 

### 5.4. Wounds Infections

**Figure 3:**
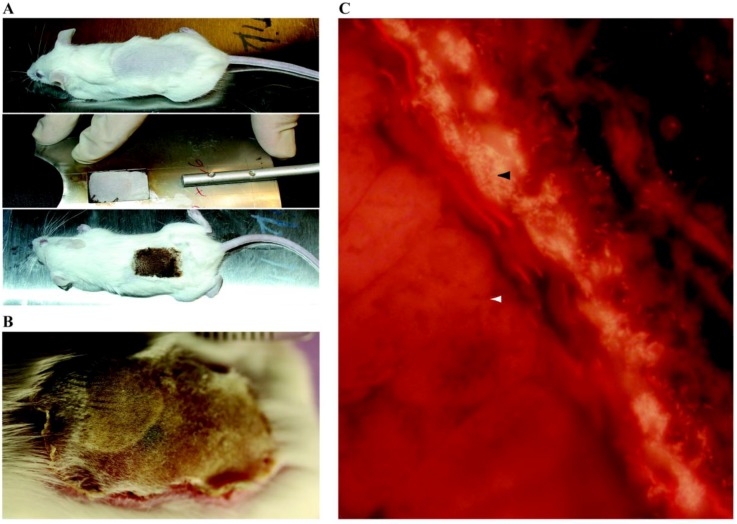
Burn wound infection biofilm in mice model. **A.** Experimental Settings. Mice are subcutaneously anaesthetized, shaved and then covered with a fire blanket and a metal plate with a window corresponding to approximately 6% of total body surface. A third-degree burn is then induced using a hot-air blower for 7 s at 330 °C. Afterwards, mice receive fluid replacement and pain therapy during the whole experiment. Lastly, mice are infected by alginate embedded *Pseudomonas aeruginosa* beneath the burn wound 2–4 days after burn wound infliction. **B.** Clinical result 4 days after the procedure. Thermal third degree lesion associated with a wound infection. **C.** Confocal laser scanning microscopy of burn wound. A slide of the wound removed *in toto* is stained with *P. aeruginosa* specific peptide nucleic acid (PNA) fluorescence *in situ* hybridization (FISH) probe (magnification × 400). *P. aeruginosa* forms dense bacterial clusters (black arrowhead) on the surface of the burn wound. White arrowhead indicates subcutaneous area. Images Credit: C. Moser, K. Thomsen, H. Calum and H. Trøstrup, Department of Clinical Microbiology, Rigshospitalet, Denmark. Adapted from [154,155].

Despite the controversial role of biofilms in delayed wound healing, it is now well accepted that wounds are colonized by biofilms as confirmed by SEM and other molecular techniques [148,149,150,151]. The most commonly studied microorganisms associated with wounds infections are *S. aureus* and *P. aeruginosa*. Several animal models of traumatic wounds including skin abrasions, burns, lacerations, surgical and excisional wounds or open fractures have been published. Dai *et al.*, developed a needle scratch mouse model in which they used genetically engineered bioluminescent *S. aureus* to study biofilm formation and the use of photodynamic therapy [152]. Several burn models have been developed [150,153], amongst which is a mouse model that allowed the visualization of *P. aeruginosa* biofilms infection in a third degree lesion using PNA-FISH (Figure 3) [154]. 

Besides, other animal models were developed to study the involvement of different bacteria, such as *S. aureus, S. epidermidis, P. aeruginosa* and *E. coli* in excisional wound infections and allowed to (i) show the infiltration of inflammatory cells within *S. aureus* clusters, (ii) confirm the presence of “membrane like structures” by electron microscopy (iii) reveal the important role neutrophils play in host defense and (iv) assess bacterial susceptibility to antibiotic therapy [156]. In addition, a cutaneous porcine wound model allowed the evaluation of topical antimicrobial treatment of *S. aureus* biofilms [157]. *In vivo* murine models also allowed deciphering the effect of RNAIII-inhibiting peptide (RIP, a quorum sensing inhibitor) in combination with teicoplanin against methicillin-resistant *S. aureus* [158] and to directly correlate *P. aeruginosa* autoinducer with tissue destruction and inflammatory response using pressure-induced ischemic wound model in rats [159]. Diabetic patients are especially prone to develop problematic wound infections that are colonized by several species of microbes forming complex multispecies biofilms of up to 1600 different microorganisms [149,160]. Although animal models are scarce, a leptin receptor-deficient mice model was developed to study synergistic effects between *E. coli, Bacteroides fragilis* and *Clostridium perfringens* in type 2 diabetes foot wounds [161]. 

### 5.5. Endocarditis

**Figure 4:**
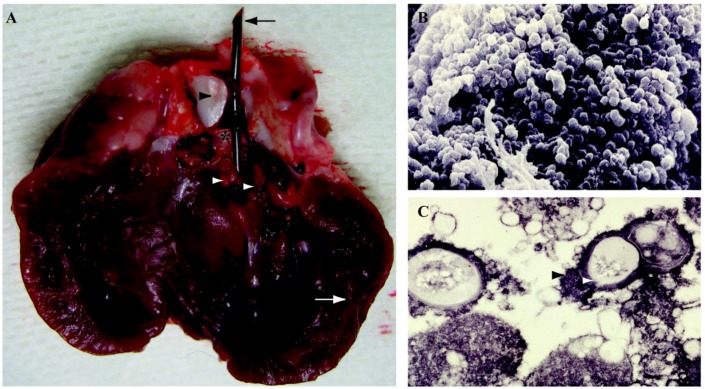
Native valve endocarditis in rabbit model. **A.** Post-mortem examination of a rabbit heart. Aortic endocarditis is induced in female New Zealand White rabbits by insertion of a polyethylene catheter (black arrow) through the right carotid artery into the left ventricle. Twenty-four hours after catheter insertion, pathogenic bacteria were inoculated through ear vein in each rabbit. The catheter is left in place throughout the experiment. Animals are killed 8 h after the last antibiotic injection and the vegetations (white arrowheads) from each rabbit are excised, rinsed in saline, pooled, and weighed. White arrow: left ventricle wall; black arrowhead: aorta; black star: aortic valve. **B.** Scanning electron microscopy of vegetation after 11 days of infection. Biofilm formed by *Streptococcus* spp. at the surface of native aortic valve. **C.** Transmission electron microscopy of bacteria from vegetation after 11 days of infection. Ruthenium red staining reveals the presence of an extracellular matrix (black arrowhead) surrounding *Streptococcus* spp. (white arrowhead) causing native aortic endocarditis. Images credit: A.-C. Crémieux (EA3647, Université Versailles Saint-Quentin), V. Dubée and B. Fantin (EA3964, Université Paris Diderot, Faculté de Médecine, Paris, France). Adapted from [167,168].

In the early 70s, in order to study infective endocarditis (IE), or the infection of heart inner lining, experimental models using rabbits, rats, pigs and cats were developed. However, the fact that progression of IE is due to biofilm establishment was demonstrated in the early 2000s [162,163]. Although, several microorganims have been associated with IE, including streptococci, staphylococci, and *Candida* [164,165], *S. aureus* is the major cause of endocarditis and thus, has been extensively studied in experimental IE. Rabbit endocarditis model has been used to decipher genetics of *S. aureus* biofilms *in vivo*, for instance, the positive modulation of the alpha-toxin gene (*hla*) by *agr*, *sarA* and *sae*, three major *S. aureus* global regulators [166]. In addition, the rabbit model allowed the study of streptococcal endocarditis confirming the presence of a biofilm structure by electron microscopy (Figure 4) [167,168]. A rat model of IE was also developed wherein endocarditis was induced by continuous low-grade bacteremia mimicking spontaneous bacteremia in humans [169]. This model was later used to highlight the role of *S. aureus* adhesion to fibrinogen and fibronectin, as well as platelet aggregation, in the initiation of *in vivo* IE [170]. Several other animal studies have been performed to evaluate the efficiency of single or combination therapies against IE with oritavancin, vancomycin, gentamicin, daptomycin or ceftobiprole [171,172,173].

### 5.6. Ear, Nose, Throat Infections

#### 5.6.1. Chronic Otitis Media (OM)

OM is the infection of middle ear leading to inflammation and is one of the most common diseases affecting children. First evidence of biofilm involvement was confirmed by direct detection in mucosa samples recovered from middle ears of children with chronic and recurrent OM [174]. Three major pathogenic agents of OM, *S. pneumoniae*, nontypeable *H. influenzae* (NTHI) and *M. catarrhalis* formed biofilms *in vivo* [175]. The most commonly used models for OM are chinchilla, mouse and rats [176,177,178]. The first chinchilla model was developed in 1976 using *S. pneumoniae* and was later used to study pathogenesis, immune response, efficacy of antimicrobials against *S. pneumoniae* and vaccine candidates [179,180,181]. Chinchilla has several advantages such as long life span of 15 years, strong ear anatomical similarity to humans, intact organ systems and immune functions, presence of large cephaloid bulla that make sampling easier and sufficient [176,182]. Moreover, this model enables direct visualization of biofilm development on the middle ear mucosa by CLSM [183,184]. Amongst other reports, this model showed that group A streptococcus (GAS) biofilms are inhibited in absence of virulence regulator Srv [185] and that c-di-GMP improves persistence of *P. aeruginosa* biofilms in chronic supportive OM [186].

Murine models of OM have also contributed to better understanding virulence factors, bacterial adhesion, invasion mechanisms, and general or specific inflammatory responses [178,187]. For instance, the use of Swiss-webster mice highlighted the role of pneumococcal proteins PavA, UspA or (Usp) A2 in adhesion [188,189]. 

In order to study long-term chronic OM, several models were adapted, for example, the primate model in cyanomolgus macaques to study the *P. aeruginosa* biofilms [190], a rat model that allowed follow up of *S. pneumoniae* up to 7 months [191] or plasminogen (plg)-deficient mice to study spontaneous development of chronic OM with varying inflammatory responses over a period of 18 weeks [192].

#### 5.6.2. Chronic Rhinosinusitis (CRS)

Chronic rhinosinusitis is the inflammation of paranasal sinuses, mainly due to bacterial invasion, typically of *S. aureus*, CoNS (coagulase negative staphylococci), *P. aeruginosa*, *S. pneumoniae* or *H. influenzae.* Several clinical studies demonstrated biofilm morphology in mucous samples from human CRS [193,194]. The most common CRS model is rabbit which allows animals monitoring up to 9 months for signs of inflammation [195,196] but mouse and sheep models have also been developed [197,198,199].

### 5.7. Dental Biofilms

Dental plaque is one of the most common types of polymicrobial biofilms that develop on susceptible tooth surfaces [200,201,202].

#### 5.7.1. Dental Caries

The main factors governing the virulence of the dental caries pathogenicity are extracellular polysaccharide (EPS) matrix and acidified plaque milieu [203]. Several models have been developed to study the cariogenic biofilms, amongst which, the most widely used is the rodent model of cariogenesis using streptococci. It has enabled us to define the infectious character of the disease, role of different genes in cariogenic process and the effect of salivary proteins in plaque formation [204,205,206]. Topical administration of bacteria to study dental plaques has been carried out in Sprague Dawley rats and there is evidence of different outcomes in host response depending on genetic background of rats [207,208]. Furthermore, this model has been used to evaluate antimicrobials against dental plaques [209,210].

#### 5.7.2. Periodontitis

Periodontitis, or destruction of periodontium structure, is due to the presence of pathogenic biofilms on the gingival and periodontal tissues resulting in heightened inflammatory response. It involves both innate and acquired immunity [211,212]. The major pathogens causing supra and subgingival biofilms are Gram-negative anaerobes such as *Porphyromonas gingivalis*, *Treponema denticola* and *Tannerella forsythia,* referred to in humans as “red complex” [213]. Many models of periodontitis have been developed using primates, dogs, rodents, rabbits, pigs and ferrets [214,215]. Nevertheless, the use of superior mammals has ethical, handling, housing and expense related issues [216,217,218]. Thus, their use should be confined to preclinical studies. Rodents have dental gingival area similar to humans [219], and periodontitis disease was also shown to induce bone loss in these animals [220,221]. Hence, use of rodents is relevant to study microbiological and immunological aspects associated with periodontitis [212,222]. For example, it has been shown that *T. denticola* persisted in a mice gavage model up to 71 days and significantly reduced IL10 [223] whereas *P. gingivalis* could be detected up to 11 weeks with migration of monocytes and neutrophils in gingival connective tissues [207]. Several studies in rodents have also addressed synergistic effects of such polymicrobial infections in periodontitis outcome [200,222,224]. Finally, an experimental rabbit model was used to evaluate efficiency of different anti-inflammatory molecules and antimicrobials such as resolvins, protectins, lipoxins in periodontitis induced by *P. gingivalis* [225].

### 5.8. Other Biofilm-Related Infections

#### 5.8.1. Osteomyelitis

Osteomyelitis, or the infection of the bones or bone marrow, is a frequent complication associated with open fractures [226]. Direct scanning electron microscopy confirmed the presence of bacterial biofilms in osteomyelitic bone samples [227,228]. Since the first study in 1941 of *S. aureus* chronic osteomyelitis in tibia [229], the rabbit model has been widely used to study chronic bone infection. This has allowed to identify proteins involved in *S. aureus* biofilm development in bones, and to evaluate the effect of various antimicrobials [230,231,232]. In another study, an experimental rat model of MRSA osteomyelitis was used to demonstrate the superiority of fosfomycin over daptomycin in treating osteomyelitis [233]. More recently, a non-invasive mouse model using bioluminescence allowed monitoring of chronic femur *S. aureus* infection up to 21 days [234].

## 6. *In vivo* Models of Device-Related Infections

The first evidence of the involvement of biofilm in device-related infections was provided in 1982 with electron microscopy study of a pacemaker implanted in a patient with recurrent *S. aureus* bloodstream infection [235]. Since then, almost all types of indwelling devices have been associated with the occurrence of bacterial or fungal biofilms (Figure 1) [162]. Because of a high tolerance towards antibiotics, these device-related infections are difficult to treat and expose patients to the risk of recurrence [236]. As the role of biofilm has been increasingly recognized, many *in vivo* models of device-related infections have been developed to validate *in vitro* data regarding mechanisticquestions, as well as to assess the preventive or curative approaches specifically targeting biofilm lifestyles [237,238,239,240] (Table 3). 

*In vivo* models of device-related infections can be broadly divided in two groups. On the one hand, the foreign-body is inserted in the organ or in the same position as it is used in clinics. Examples of these “site-specific models” are intravascular catheter models or intrafemoral pins or wires. On the other hand, the foreign-body is inserted in a subcutaneous pocket so that there is no contact with a specific structure or organ, therefore defining the “subcutaneous models” such as tissue cage model or subcutaneously inserted pieces of catheters.

### 6.1. Site-Specific Models

#### 6.1.1. Vascular Catheters

During an outbreak of catheter-related infections, the observation that *S. epidermidis* was able to produce extracellular matrix (in the past called “slime”) and adhere to surfaces identified the importance of biofilm in this setting [241,242]. First attempts to set-up *in vivo* models relied on the subcutaneous implantation of catheter cut in pieces (see the “subcutaneous models” section) [237]. Even though this approach helped understanding mechanisms involved in *S. epidermidis* biofilm formation, these models lacked several important features of catheter-related infections such as interaction with blood components or blood flow [243]. These limitations led to considerable efforts to develop *in vivo* models integrating these aspects.

The most popular intravascular venous catheter model developed to study bacterial colonization and subsequent infection was described in 1999 using a silastic catheter inserted into the superior vena cava of adult rats [239]. The rat model has been used to validate the *in vivo* importance of polysaccharide intercellular adhesin (PIA) and autolysin AtlE in the establishment of a *S. epidermidis* biofilm and in its pathogenesis (bloodstream infection and metastatic disease) [239,244]. This model allowed assessing preventive approaches, such as use of RNAIII-inhibiting peptide (RIP) [245] or vaccination with immunization of rats prior to catheter insertion leading to a protective effect towards bacterial colonization of the device by *S. aureus* or *S. epidermidis* [246]. Other animals such as rabbits or mice have also been used to develop such central venous catheter (CVC) models with similar surgical procedures [247,248].

**Figure 5:**
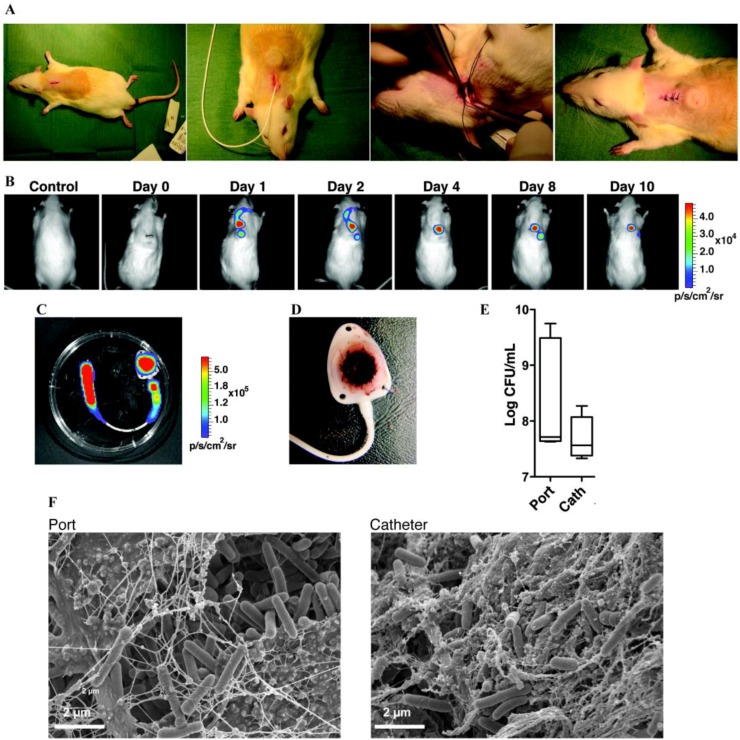
Totally implantable venous access port (TIVAP)-associated biofilm using rat model. **A.** Experimental Settings. Rats are anesthetized and shaved before starting the procedure. After skin disinfection, an incision is made at the dorsal midline, a subcutaneous pocket is created and the port is carefully inserted before being held intact by sutures. An incision is made in the neck area on the ventral side in order to access the external jugular vein. The catheter is inserted into the vein and pushed up to the superior vena cava. Suturing of both the dorsal and ventral sides closed the wounds and rats received analgesia at the end of the experiment. **B.** Monitoring of TIVAP colonization by *E. coli*. Five days after TIVAP insertion, 10^4^ CFU of *E. coli* in 100 µL are injected into the port and photon emission is measured over a period of 10 days to monitor biofilm growth. Dorsal view of a representative rat, showing progression of biofilm signals towards the catheter tip and then restriction to the port. **C**, **D**, **E** and **F**. Bacterial colonization of TIVAP leads to biofilm formation. Rats are sacrificed 10 days post-infection and TIVAP are removed aseptically for examination. **C.** Photon emission of the removed TIVAP colonized by *E. coli* biofilm. **D.** Macroscopic examination after septum removal showing blood clots and deposits inside the port. **E.** Bacterial cells are harvested from the catheter and port separately and plated on LB agar for CFU counting. **F.** SEM images confirming biofilm formation in TIVAP *in vivo* in the port and catheter. Adapted from [256].

Attempts to eradicate *in vivo* biofilms have been made using antibiotic lock technique/therapy (ALT). In this treatment, a highly concentrated antibiotic solution is allowed to dwell for 12 to 24 hours inside the lumen of a catheter. Conclusions of the different studies are difficult to compare as different authors used various compounds, concentrations and time of treatment. It was demonstrated that against *S. aureus*, quinupristin-dalfopristin, linezolid, vancomycin, and ciprofloxacin dwelling for 1 hour were able to reduce the amount of survival CFU in blood and on catheter tip but none of them eradicated biofilm, suggesting the importance of a longer dwelling time [249]. ALT composed of daptomycin associated with systemic treatment was also assessed in order to eradicate *S. aureus* and *S. epidermidis* catheter-related infections using polyurethane CVC: vancomycin and daptomycin were equally efficient against methicillin-resistant *S. epidermidis* (MRSE) infection [250]. A rabbit CVC model allowed researchers to demonstrate that heparin had no effect and that it did not improve the efficacy of vancomycin or ciprofloxacin ALT on *S. aureus* biofilm [251]. Later, the same group compared linezolid, vancomycin, gentamicin and ciprofloxacin antibiotic solutions as ALT against *S. aureus* biofilm. In this model, gentamicin (40 mg/mL) was the most efficient drug against methicillin-susceptible *S. aureus* (MSSA) and methicillin-resistant *S. aureus* (MRSA) [252]. In addition, association of minocycline-EDTA as lock therapy has been shown to efficiently reduce the number of rabbits with positive blood cultures and in eradicating biofilm on the catheter tip [253]. All these data have been used to design clinical studies and some of these compounds are now used in clinics [254,255].

Recently, we developed an *in vivo* model of totally implantable venous access port (TIVAP)-related infection to study long-term biofilms (Figure 5) [256]. TIVAPs are used for antineoplastic chemotherapy, parenteral nutrition or blood products in humans. These devices are composed of a vascular catheter inserted into the external jugular vein of a rat connected to subcutaneous port. This model does not require the use of a restraint jacket and use of bioluminescent strains allows long-term non-invasive monitoring of biofilm formation. We used this model to demonstrate the ability of an association of gentamicin and tetrasodium EDTA to eradicate 3-day old biofilm of *S. epidermidis*, *S. aureus* (MRSA or MSSA), *P. aeruginosa* and *E. coli* [257]. A clinical study can now be set-up, based on these data.

#### 6.1.2. Urinary Catheters

The observation that systemic antibiotic failed to eradicate bacteriuria without removal of the urinary tract device was made decades ago [258]. In 1985, the electron microscopic study of a urethral catheter removed due to relapsing catheter associated urinary tract infection (CAUTI) revealed the presence of a bacterial biofilm [259]. Since then, many *in vivo* models have been developed in order to mimic these situations. Most of them rely on the insertion of a foreign-body inside the bladder and may or may not include a surgical procedure. Therefore, urinary catheter-associated models can be classified in 3 groups: (i) and (ii) foreign-body left inside the bladder with or without a surgical procedure; (iii) a complete urinary catheter with a tip located inside the bladder, associated with a urine collection system and exiting through urethra.

(i) Surgical models. These models rely on the surgical insertion of a foreign-body inside an animal bladder such as glass beads [260] or pieces of urinary catheter [261]. They allowed the study the effect of persistent bladder colonization on renal scaring [260] but also to assess different therapeutic strategies [260,261]. First develop in rats, the same model was later adapted in mice to demonstrate the use of mannitol as an adjuvant to gentamicin to eradicate biofilm persisters of *E. coli,* which can be a promising clinical candidate [262].

(ii) Non-surgical models. The principle of these models is to transurethrally insert pieces of catheter inside the animal bladder [263]. These models have been developed in rats, rabbits and mice to study several aspects of CAUTI such as bladder inflammation [263], virulence of *E. faecalis* on a silicone implant [264] or preventive approaches such as Triclosan^®^ (a broad spectrum antiseptic) [265]. Various curative treatments have been assessed in these models, such as antibiotics alone [266] or in the association with small molecules (like mannosides) to prevent CAUTI [267]. This adjuvant approach still needs to be validated in clinic.

(iii) Models of externalized urinary catheters. In order to mimic a complete urinary catheter with an externalized system, urethral models were developed in rabbits and sheep and used to study the effect of systemic antibiotics on viable cell count or biofilm structure on the catheter tip [268,269]. Preventive approaches have also been assessed such as antiseptic-coated catheters like Gendine [270] or Low-Energy Surface Acoustic Waves [271]. Another approach was developed using a sheep model to study iontophoresis as a preventive measure [272]. In this model, urethral catheters are modified in order to deliver a current to electrodes located on the catheter tip leading to the production of ions of soluble salts and allowing the formation of local biocide. After 20 to 21 days, this approach significantly reduced bacterial burden in urine. 

Even though all these models identified promising compounds, clinical studies remain rare or gave negative results such as a recent clinical study using antibiotic-coated catheters without any significant clinical benefit [273]. 

#### 6.1.3. Orthopedic Implants and Prosthetic Joints

First reported work trying to reproduce osteomyelitis in rabbits was published as early as 1885 [274]. In early 70’s, it was shown that the presence of a foreign-body inserted into a rabbit tibia (stainless steel pin) could potentiate the development of chronic *S. aureus* osteomyelitis [275]. Interestingly, it is only ten years later that the link between biofilm formation and orthopedic devices was confirmed in clinics and using a rabbit model of foreign-body inserted inside the tibia [276,277]. Even though rabbits have been frequently used for these studies, orthopedic implant models have been developed in many other animals with a wide range of foreign-bodies and sites of insertion (see Table 3). These models allowed the study of the influence of various types of materials such as stainless steel, titanium, cobalt chromium, polyethylene or polymethylmetacrylate on bacterial adhesion [278,279]. It has also been demonstrated that bone devascularization, presence of foreign-body and bacterial virulence played a key role in the incidence of osteomyelitis [280]. Moreover, the impact of adaptive immune responses was studied to show that Th2/Treg responses played a key protective role against chronic *S. aureus* implant infection [281]. Besides, preventive approaches have been assessed, such as use of gentamicin for systemic and local treatment or as a coating of the device [282,283]. Lastly, different models have been developed to study antibiotic-impregnated cement or implants [284]. Promising results were obtained using gentamicin-coated nails in the tibia in a clinical pilot study [285].

Even though osteomyelitis and prosthetic joint infections (PJI) share common features, different animal models have been developed in order to assess specific characteristics of PJI. These models allowed to study diagnostic procedures such as magnetic resonance imaging (MRI) modification after *S. aureus* PJI [286] as well as preventive approaches like gentamicin-loaded bone cement for prevention of *E. coli* PJI [287] or prevention of *S. aureus* PJI after bloodstream infection [288]. Another preventive approach relying on a minocycline/rifampin-impregnated bioresorbable polymer implant coating has been shown to reduce biofilm formation [289]. Another key contribution of these models is the study of distribution and efficacy of frequently used antibiotics such as fluoroquinolones [290]. 

#### 6.1.4. Endotracheal Tubes

**Figure 6:**
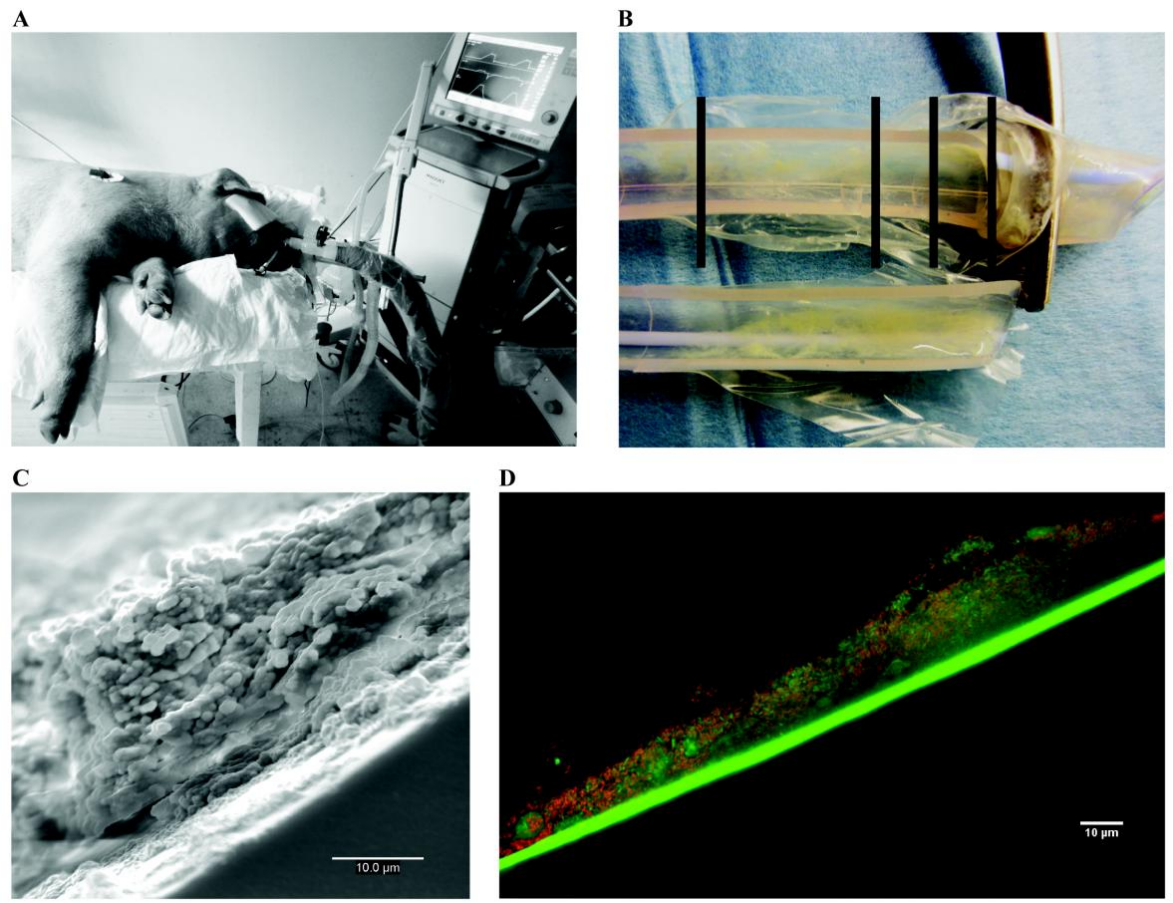
Model of endotracheal tube biofilm–associated infections in ventilated pigs. **A.** Experimental Settings. Large-White Landrace female pig (36 Kg) orotracheally intubated and mechanically ventilated. Following intubation, the animal received an oropharyngeal challenge of *Pseudomonas aeruginosa*. During mechanical ventilation, endogenous oropharyngeal bacteria and *Pseudomonas aeruginosa* rapidly colonize the internal surface of the endotracheal tube. Bacteria within the endotracheal tube constitute a persistent source of pathogens, which may result in ventilator-associated tracheobronchitis and pneumonia. **B.** Endotracheal tube internal surface following 72 hours of mechanical ventilation. After extubation, the endotracheal tube external surface is cleaned with sterile gauzes and decontaminated by careful rinsing with 80% alcohol and saline solution and then longitudinally sliced open. Two 1 cm long sections and one 3 cm long section of the dependent half of endotracheal tube are dissected for confocal electron microscopy, scanning electron microscopy and quantitative microbiological studies, respectively. **C.** Scanning electron microscopy (magnification × 2000) of the internal surface of endotracheal tube (lateral view). Bacterial communities are adherent to the endotracheal tube surface and surrounded by the extracellular matrix. **D.** Confocal laser scanning microscopy of the internal surface of endotracheal tube (lateral view). The lumen of endotracheal tube is stained with BacLight Live/Dead stain (magnification × 250). *Pseudomonas aeruginosa* biofilm is adherent on the internal surface of endotracheal tube. Eukaryotic cells are also present within the biomass. Images Credit: G. Li Bassi and L. Fernandez-Barat, Pulmonary and Critical Care Unit, Division of Animal Experimentation, Hospital Clinic, Barcelona. Adapted from [294,299].

Biofilm formation in endotracheal tubes was described in 1989 [291] and it is now considered that they can constitute a potential source of ventilator-associated pneumonia (VAP) that can escape antibiotics in case of systemic treatment [292]. Animal models using sheep, pigs or dogs have been developed. They rely on the orotracheal intubation of animals with endotracheal tubes and mechanical ventilation for 24 to 96 hours and have been used to study preventive or curative strategies (see Figure 6) [293,294,295]. These models have, for example, been used to demonstrate the effectiveness of endotracheal tubes coated with silver-sulfadiazine/chlorhexidine in polyurethane or silver hydrogel coating to reduce bacterial colonization [293,295]. These data enabled the performance of a clinical study demonstrating that this approach significantly reduced the incidence of VAP in clinics [296]. This model was later used to show that a mechanical removal of biofilm from the surface of endotracheal tubes prolonged the bactericidal activity of such coated devices. This approach called “mucus shaver” allows the retention of the device in place and biofilm removal from its surface [297]. This mucus shaver procedure was assessed in a pilot study including 12 patients in each group and allowed a significant reduction of the number of endotracheal tubes colonized with biofilm [298]. 

#### 6.1.5. Vascular Grafts

Identification of involvement of biofilm in the occurrence of vascular graft-related infection was made at the end of the 80’s [300]. Such graft-related infection was later studied in a dog model to develop diagnostic or surgical procedures [300]. This model relied on the implantation of dacron prosthesis colonized by *S. epidermidis* on the infrarenal aorta of a dog leading to the development of a prosthetic vascular graft infection (PVGI). This model was notably used to study the effectiveness of rifampicin-bonded gelatin-impregnated grafts in reducing the number of animals with persistent biofilm colonization [301]. Another group confirmed that rifampicin-soaked silver-coated polyester (RSSCP) was more efficient than expanded polytetrafluoroethylene (ePTFE) graft replacements, in the treatment of aortic PVGI porcine model [302]. This model was also adapted in miniature pigs to demonstrate that Dacron graft bonded with chlorhexidine, rifampicin, and minocycline was a good candidate for the prevention of *S. aureus* related infections [303]. First clinical studies using antibiotic-impregnated grafts gave promising results in humans but still need to be confirmed by comparative trials [304].

#### 6.1.6. Tissue Fillers

In 1992, study of removed silicone devices, such as mammary implants or tissue expanders led to the identification of bacterial biofilm on the surface of these foreign-bodies [305]. The development of biofilms was associated with the occurrence of capsular contraction, a late complication of such foreign-bodies. A porcine model has been used to study biofilm formation on the surface of breast implants [306,307]. Implants were inserted with or without a circular disk of minocycline-rifampicin-impregnated polypropylene mesh and left in place for 16 weeks [306]. This preventive approach reduced the incidence of capsular contracture and biofilm formation. 

#### 6.1.7. Contact Lenses

Identification of contact lens as a suitable surface for bacterial adhesion and biofilm formation was made at the end of 80s [308]. Biofilm colonization has also been demonstrated to be associated with the occurrence of corneal ulceration [309]. Several animal models have been developed to study contact lenses tolerance or the influence of bacterial inoculation on the incidence of keratitis such as rabbit model or guinea pig model [310]. While these models have never been used to specifically address questions regarding bacterial biofilms, a specific mouse model has been developed in 2010 in order to study fungal biofilms on contact lenses and its link to keratitis [311].

#### 6.1.8. Dental Implants

Confocal laser scanning microscopic studies of oral implants confirmed that biofilm was present with a structure close to what is seen in dental plaque [312]. It has also been shown that the development of these dental implant-related biofilms could play a role in periimplant infections (also called periimplantitis) and progressive bone loss around the implant. The first animal model of dental implant-associated infections was developed in 1992 involving the replacement of mandibular right premolars by complete titanium implants [313]. In this model, mucosal lesions around implant-associated plaque or teeth-associated plaque were comparable but bacterial biofilm *per se* has not been studied [314]. However, a rat model has been developed to specifically study the effect of *Aggregatibacter actinomycetemcomitans* biofilm [315].

Aside from these animal models, different groups developed human models. All these human models rely on the positioning of a fixation system applied on the mandibular region, inside the mouth of healthy volunteers on which disks made of various surfaces can be placed. For instance, this approach has been used to study the impact of surface roughness on plaque colonization of titanium device [316]. Another group used this approach to demonstrate that bacterial adhesion on implant surfaces was significantly lower with zirconium oxide surface as compared with pure titanium [317]. 

### 6.2. Subcutaneous Foreign-Body Models

#### 6.2.1. Tissue Cage

These models were developed in the early 80’s to mimic a foreign-body-related infection [240]. These cages consist in rigid tubes with perforations and sealed at each end. These devices can be implanted in mice, rats, hamsters, guinea pigs [240] and ponies. Bacterial contamination can be made before or after tissue cage insertion by a percutaneous injection inside the tissue cage. This model has been used to study different aspects ranging from the influence of host immune response to the effectiveness of different treatments [318]. For instance, the efficacy of adjunction of rifampicin in treatment regimen was suggested in tissue cage models before being confirmed in clinical studies [319]. Fosfomycin and daptomycin are currently being investigated and could be promising candidates against foreign-body-related infections [318]. For an extensive review of these models, see [22,320].

#### 6.2.2. Vascular Catheters

This model has mostly been developed using mice and involves the insertion of a 1-cm segment of vascular catheter under the skin, in a subcutaneous space [243]. The biomaterial can be contaminated by bacteria before or after the surgical procedure and various biomaterials have been used including Teflon^®^, polyurethane, silastic, latex, dacron, Gortex^®^ [243]. This model was, for example, used to study the importance of staphylococcal accessory regulator (*sarA*) and *ica* for *S. aureus in vivo* biofilm formation [321]. Another group recently demonstrated that there was a reduction in cytokine production during biofilm formation and limited macrophage invasion into *S. aureus* biofilms *in vivo* [322]. This model can be used to assess therapeutic options in order to mimic ALT with the injection of antibiotic inside the lumen of subcutaneous catheters [323]. Preventive approaches can also be assessed like the use of cerium nitrate, chitosan and hamamelitannin to prevent the formation of biofilm of *S. epidermidis*, *S. aureus*, *Acinetobacter baumannii* or *C. albicans* [324]. Many authors use bioluminescent variants of bacteria to follow colonization non-invasively [325]. Using a rabbit model of subcutaneous catheters, minocycline and rifampin coating prevented the colonization by *S. aureus* [326], which later was demonstrated to be more efficient than chlorhexidine+silver sulfadiazine coated vascular catheters in a human clinical trial [327]. Other vascular catheter coatings have been studied such as triclosan and dispersin B (an antibiofilm enzyme) to prevent *S. aureus* colonization [328]. 

#### 6.2.3. Other Subcutaneous Models

*Bone cement.* A study comparing two commercial acrylic bone cements shaped like a disk demonstrated that association of gentamicin-loaded cement and pulsed ultrasound reduced by 50% the number of viable bacteria recovered from the surface of the implant after sacrifice [329].

*Pacing device.* A rabbit model has been developed to study biofilm formation on the surface of a pacing device, which demonstrated that the use of a mesh envelope incorporating minocycline and rifampicin around the device prevented biofilm formation of *S. epidermidis*, *S. capitis*, *E. coli* and *A. baumannii* [330].

*Cardiac valves.* To study cardiac valve-related infections, *in vivo* models were developed in guinea pigs [331], mice [332] and rabbits [333]. This type of studies focused on the use of silver-coated polyester fabric [331] or minocycline/rifampin coated sewing cuffs to prevent bacterial colonization and infection [334].

*Vascular grafts*. Different models were developed in hamster [335], mice and rats [336]. These models have been used to study preventive approaches such as antibiotic bonded grafts or vancomycin delivered from glycerylmonostearate (GMS) implants [336]. Different curative approaches have been studied using these models including intraabscess urokinase associated with systemic gentamicin [335] or quorum-sensing inhibitor FS3-coated vascular graft associated with daptomycin [337].

*Polyethylene disks*. Subcutaneous implantation of polyethylene disks in mice or rabbits showed promising effects of ultrasound combined with systemic gentamicin against *E. coli* [338]. 

*Beads*. Polymethylmethacrylate beads loaded with or without the quorum-sensing inhibitor RIP associated with or without vancomycin were inserted in subcutaneous space of rats. This approach efficiently prevented *S. aureus* biofilm formation [339]. 

*Surgical meshes*. This model involves the insertion of resorbable or non resorbable surgical meshes colonized by *S. aureus* biofilm inside subcutaneous pockets [340]. While biofilm persisted around non-degradable meshes up to 28 days, bacteria disappeared from surrounding tissues in case of degradable mesh [341]. 

## 7. Take-Home Messages and Future Directions

### 7.1. Pitfalls of *in vivo* Biofilm-Related Infection Models

As discussed in this review, use of numerous *in vitro* and *in vivo* models in the last twenty years has provided massive information on most human biofilm-related infections, even though models dedicated to some biofilm-related infections are missing (see Table 4). Numerous *in vivo* models have been developed for each infectious disease to address specific questions regarding initial adhesion, assessment of different surfaces, preventive or curative approaches, as illustrated by the large number of models used to study CVC-related infections [243,248,256]. Therefore, there is no “gold-standard” as each model may provide an answer to a specific question, depending on host immune system, size or surface of the device and environment. These multiple models provide researchers with a myriad of options in order to choose the more appropriate model that will answer the biological question raised. However, a concerted effort is needed to standardize studies using identical models (in term of animal lineages, route and dose of inoculation, *etc*.) so that scientists using the same model can compare their results. For instance, among studies dealing with ALT for CVC-related infections, several drugs were studied at different concentrations or dwelling times, therefore, impeding any comparisons between results [249,251,252]. In other fields, scientists have proposed guidelines to standardize assays and their interpretation in order to homogenize their results (see for instance [342]). Such an effort should be made in *in vivo* modeling of biofilm-related infections.

**Table 4:** Biofilm-related infections without specific *in vivo* model. Among them, some diseases have *in vivo* models but without any application for biofilm studies.

Type of biofilm-related infection	Animal model for the disease (References)	Implication of biofilms in clinics (References)
Tissue-related infections		
Keratitis	Yes [446]	Yes [447]
Endophthalmitis	Yes [448]	Yes [449]
Chronic tonsillitis	Yes [450]	Yes [451]
Chronic laryngitis	No	Yes [452]
Bacterial Vaginosis	Yes [453]	Yes, reviewed [454]
Meningitidis	Yes [455]	Yes, discussed and reviewed in [456]
Device related-infections		
Cochlear implants	No	Yes [457]
Voice prosthesis	No	Yes [458]
Neurological devices	No	Yes, reviewed in [459]
Penile prosthesis	No	Yes, reviewed in [460]
Biliary stent	Yes [461]	Yes [462]

Furthermore, many concerns have been raised regarding the scarce translation from *in vitro* and *in vivo* models to clinical studies [343]. This drawback is not limited to the field of microbiology but probably reflects limitations of *in vivo* studies [344]. First, obviously, differences exist between humans and animals used for *in vivo* studies, especially response towards microorganisms or their components. A striking example of the latter is the difference between human and murine sensitivity to LPS that may distort conclusions of studies relying only on this model [345]. Secondly, the choice of animal for *in vivo* studies is frequently based on experimental convenience and rarely include environmental factors that have been shown to have important influence on the outcome of an infection [346]. The same can be argued for the choice of bacterial strains that may not be representative of the natural setting. One well-known example is the use of non-mucoid PAO1 *P. aeruginosa* laboratory strain that causes acute types of infection, which are not representative of the clinical symptoms in CF chronic infection. Lastly, as for other *in vivo* studies, rigorous statistical analysis and experimental set-up are mandatory in order to avoid any false positive interpretation [347]. One can foresee that recent publication of new guidelines for reporting animal research will improve quality of experimental *in vivo* models [346,348].

### 7.2. Under-Developed Aspects of Biofilm-Related Infections

#### 7.2.1. Synergy between Biofilm Tolerance and Resistance Genes and Their Impact on Nosocomial Infections

One major challenge of biofilm research is to understand and tackle the increased tolerance of biofilms towards antimicrobial agents including antibiotics [236]. This ability to endure high concentration of antibiotics complicates treatment of biofilm-related infections and is a threat that is enhanced by microorganisms carrying resistance genes such as extended spectrum β-lactamase (ESBL) or methicillin-resistance. Increased frequency of horizontal transfers such as conjugation, transformation or transduction has been demonstrated *in vitro* within biofilms [349]. Hence, biofilms may also be expected to facilitate the transfer of resistance genes as demonstrated in an *in vitro* study with an increased rate of transfer of a plasmid encoding CTX-M-15 (an ESBL) in a *K. pneumoniae* biofilm as compared to planktonic conditions [350]. Interestingly, many transmissible DNA elements encode biofilm promoting factors including various adhesins such as conjugative pili, fimbriae or autotransporter adhesins and persistence genes such as toxin/anti-toxin modules. This, therefore somehow promotes their own transfer by means of their capacity to increase biofilm formation or antibiotic tolerance. Additionally, these phenomenon may be favored by architecture of biofilms in which antibiotics reach certain areas of biofilms only at sub-inhibitory concentrations that are known to increase (i) the likelihood of selecting resistant mutants; (ii) rate of mutations; (iii) biofilm formation and (iv) gene transfer [351,352,353].

The possible interplay between biofilm tolerance, gene transfer and spread of resistance can be of key-importance in nosocomial settings but remains to be demonstrated in clinical settings or even in a relevant *in vivo* model of biofilm-related infections. Therefore, establishing *in vivo* models allowing the study of mechanisms developed by biofilm bacteria to survive antibiotics is a major challenge that should be met in the coming years.

#### 7.2.2. A Switch towards Models to Study Biofilm Polymicrobial Infections?

While environmental biofilms are known to be composed of multiple species, the concept and demonstration that human infections can arise in the context of polymicrobial biofilms has been overlooked until recently. Koch’s postulates associating one microorganism with one disease as well as the extensive use of culture-dependent isolation techniques have for long time masked the possibility that the nature of the flora among which the pathogenic bacteria are embedded can strongly influence its behavior and the infection outcome. Today, due to the advent of culture-independent methodologies, many tissue infectious diseases are likely associated with the presence of multiple microorganisms like otitis media, oral cavity diseases, vaginosis, wound infections and CF lung infections [354]. The formal demonstration of the existence of polymicrobial biofilms and their link to infection is still a matter of debate (see the reviews [354,355]). Nevertheless, several studies allowed direct visualization of polymicrobial biofilms in patients. For instance, electron microscopy studies of biofilm in chronic wounds demonstrated the presence of biofilm composed of bacteria of various morphotypes (rods and cocci) [149]. This diversity was confirmed using molecular biology tools. Specific FISH probes were also used to show that some biofilms in chronic otitis media are composed of *H. influenzae* and *S. pneumoniae* [174]. Interestingly, analyses of the flora comprising biofilms responsible for catheter associated infections like CAUTI or catheter-related bloodstream infections (CRBSI) also demonstrated the presence of multiple species [356,357]. It is therefore suspected that a large part of biofilm-related infections might be indeed linked to the presence of polymicrobial biofilms. Until now, several well-suited *in vitro* models to characterize interactions between species have been developed especially using microorganisms colonizing the oral cavity, the intestine or wound infections (see, for example [25,358,359]). Surrogate non-mammalian animal models are also used to study such polymicrobial interactions within biofilms. For instance, the behavior of *P. aeruginosa* in presence of other bacteria such as *S. aureus* and that of *A. actinomycetemcomitans* in presence of *S. gordonii* was analyzed using *D. melanogaster* [359,360]. While relatively few, several studies of polymicrobial infections using *in vivo* mammalian models have been conducted recently using otitis media, wound, UTI or CF lung models [72,360,361,362,363]. Although these studies were performed with different models and microorganisms, they showed that the colonization, infection and host response was totally different depending on single or multiple species infections with notably an increased ability to colonize or cause host damages/inflammation in co-infections. These promising results urge for rapid implementation of studies aiming not only to analyze the possible polymicrobial status of still unstudied biofilm-related infections but also focusing on development of specific polymicrobial biofilm models. These future studies will certainly help to elucidate molecular mechanisms of bacterial interactions that would ultimately lead to the potential identification of molecules or vaccines that can target polymicrobial biofilms.

#### 7.2.3. What Can We Expect from Systems Biology, Computational Biology, Ecology or Experimental Evolution?

Until now, studies of polymicrobial infections using *in vivo* mammalian models remain scarce probably due to the increased complexity to develop such experimental models and the difficulty to interpret results coming both from interactions between the microorganisms and the hosts, and between microorganisms themselves. Significant progress in this area could be made by integrating new concepts and approaches to understand better the ecology of infectious diseases such as the one causing gut disorders or leading to lung destruction in CF patients [364,365].

In these complex structured ecological systems diseases are predicted to originate from the modification of the dynamic of interactions between pathogens, commensal and the hosts systems, and to be strongly dependent on the composition of the environment. Computational modeling is a common tool used to understand many biological processes, such as epidemiological dynamics [366] and emergence of antibiotic resistance [367]. Such approach has allowed genome-scale metabolic reconstruction of several pathogens including *P. aeruginosa, S. aureus*, *Burkholderia* spp. or *Salmonella* spp. and have been used to predict the behavior of bacteria in complex *in vivo* modeled environments including biofilms leading to the identification of potential target genes or lethal specific environment conditions awaiting *in vivo* validation [368,369,370]. With metabolic multi-cells modeling, taking into account interactions between microorganisms and eukaryotic cells, should come a better understanding of potential complex interactions [368]. Efforts have been made to model various aspects or properties of biofilms such as, for instance, heterogeneity within biofilms [371] or more recently to integrate biofilm formation and development with host dynamics (for example, [372]). Indeed, aspects of evolution are also important to integrate to biofilm-related infections because outcome of the chronicity of such diseases may result from long lasting interactions and co-evolution between microorganisms and hosts. Experimental evolution is a powerful tool that in combination with high throughput sequencing technology can help decipher the genetic and molecular basis of evolutionary change [373,374]. Such an approach, that has notably been used in medicine to develop attenuated live vaccines [374], has also been successfully used to understand micro-evolution and patho-adaptative mutations of *P. aeruginosa* and *B. cenopacia*, both in patients and in *in vitro* or *in vivo* murine models, during modeled or real chronic airway infections. These long-term studies identified bacterial functions that can favor persistent life style of these pathogens and that can have important clinical implications for management of the disease progression [375,376,377,378]. These results may also apply to various biofilm-related chronic infections. Thus, specific *in vivo* models of tissue or devices-associated infections could shed light over the various selective pressures acting on biofilm physiology and promoting bacterial diversity or the response of biofilm bacteria to different antibiotic treatments.

The world of infectious diseases is today taking a major turn towards the identification and integration of the multiple parameters influencing diseases' outcome [364,365,379,380]. A better spatial and temporal understanding of dynamic interactions between the microorganisms, the hosts and their environment will open a myriad of possibilities for clinicians to perform in the future an adaptive management of human infections not solely centered on antibiotics but playing with stability of communities or resource competition. Biofilm research has its place to take in this new exciting challenge.
